# Long-term survival of asexual *Zymoseptoria tritici* spores in the environment

**DOI:** 10.1186/s12915-024-02060-3

**Published:** 2024-11-19

**Authors:** William T. Kay, Paul O’Neill, Sarah J. Gurr, Helen N. Fones

**Affiliations:** 1 Biosciences, University of Exeter, Exeter, UK; 2 Department of Biology, University of Oxford, Oxford, UK

**Keywords:** *Zymoseptoria tritici*, Nutrient depletion, Long-term survival, Metabolism, Spore survival, Rain-splash dispersal, Septoria leaf blotch, Lipids

## Abstract

**Background:**

The fungal phytopathogen *Zymoseptoria tritici*, causal agent of the economically damaging Septoria tritici blotch of wheat, is different from most foliar fungal pathogens in that its germination occurs slowly and apparently randomly after arrival on the leaf surface and is followed by a potentially prolonged period of epiphytic growth and even reproduction, during which no feeding structures are formed by the fungus. Thus, understanding the cues for germination and the mechanisms that underpin survival in low-nutrient environments could provide key new avenues for disease control.

**Results:**

In this work, we examine survival, culturability and virulence of spores following transfer from a high nutrient environment to water. We find that a sub-population of *Z. tritici* spores can survive and remain virulent for at least 7 weeks in water alone, during which time multicellular structures split to single cells. The fungus relies heavily on stored lipids; however, if cell suspensions in water are dried, the cells survive without lipid utilisation. Changes in gene expression in the first hours after suspension in water reflect adaptation to stress, while longer term starvation (7 days) induces changes particularly in primary metabolism and cytochrome P450 (CYP) gene expression. Importantly, we also found that *Z. tritici* spores are equally or better able to survive in soil as in water, and that rain-splash occurring 49 days after soil inoculation can transfer cells to wheat seedlings growing in inoculated soil and cause Septoria leaf blotch disease.

**Conclusions:**

*Z. tritici* blastospores can survive in water or soil for long periods, potentially spanning the intercrop period for UK winter wheat. They rely on internal lipid stores, with no external nutrition, and although a large proportion of spores do not survive for such an extended period, those that do remain as virulent as spores grown on rich media. Thus, *Z. tritici* has exceptional survival strategies, which are likely to be important in understanding its population genetics and in developing novel routes for Septoria leaf blotch control.

**Supplementary Information:**

The online version contains supplementary material available at 10.1186/s12915-024-02060-3.

## Background

*Zymoseptoria tritici* is an ascomycete fungus that causes the economically damaging wheat disease, Septoria tritici blotch. Despite significant research effort, open questions remain around the strategy used by this fungal pathogen to obtain nutrients [1]. When infecting wheat, it forms no feeding structures and is generally considered a ‘stealth’ pathogen—a biotroph that evades detection by its host partially through slow initial growth [2]. However, some doubt has been cast upon this lifestyle description [1, 3, 4]. Fones et al. [4] demonstrated that the heavily studied isolate IPO323 can spend over 10 days on the leaf surface prior to invasion. This period of surface dwelling is not passive, but can include hyphal extension and exploration of the leaf surface, reproduction by budding [5–7] and even the formation of biofilms [8] (pre-print). It has been shown that germination, hyphal extension and the subsequent phases of leaf infection, including leaf penetration and the formation of fruiting bodies, are all asynchronous [9]. Following an initial resting phase that lasts up to 15 days, growth on the leaf surface prior to penetration continues for between 2 and 17 days, giving a total of up to 18 days on the leaf surface under optimal conditions [9]. This long, variable period of surface survival and growth stands in sharp contrast to many other fungal plant pathogens, which, with limited energy stores in the spore, have a short time frame for leaf entry and nutrient uptake from the plant and are thus adapted to navigate the leaf surface efficiently [4, 10, 11]. These fungi have highly predictable developmental processes from the detection of a host surface to the formation of feeding structures inside the leaf; while *Z. tritici* does follow a series of predictable steps from germination to entry via stomata to colonisation of the apoplast and pycnidiation in substomatal spaces [12, 13], the timing and the extent of growth at each stage is variable [7, 9]. Thus, rather than a carefully choreographed process of germination and host invasion that is efficient and responds to host cues, *Z. tritici* presents a picture of a fungus whose germination occurs at a random time after arrival on the leaf surface, whose growth is random with respect to entry points, variable in extent and prone to deviating into alternative developmental processes such as blastosporulation or biofilm formation, whether it is on a susceptible or resistant host [4, 6, 7, 9].

These unusual epiphytic behaviours in *Z. tritici* provoke questions: primarily, how does *Z. tritici* survive these long periods in the low-nutrient epiphytic environment? Further, rain-splash is an inefficient method of finding a susceptible host, with many spores landing on non-host surfaces or soil—potentially leading to additional periods of low nutrient availability. Previously, we showed that in the first few minutes after blastospores are transferred from the rich culture medium, YPD, to water, both their virulence and culturability fall sharply, unless protected by added osmolytes [14]. We suggest that there may be similarities between this experimental immersion of blastospores in water and the environmental changes experienced during rain-splash dispersal either of pycnidiospores or of epiphytically produced blastospores. During rain-splash, the nutrient rich cirrus in which pycnidiospores are extruded from the infected leaf and which protects them from desiccation [13, 15] is diluted and the spores are therefore exposed to a rapid drop in nutrient availability and osmotic pressure. While leaf-surface produced blastospores do not have a protective cirrus, there is evidence that *Z. tritici* can undergo leaf-surface blastosporulation when growing as a biofilm, in which cells are protected by a protective extracellular matrix (ECM) [8] (pre-print), [16]. Biofilm ECMs are known to provide some functions analogous to those attributed to cirrus, including the prevention of desiccation and osmotic shock, and can act as a source of nutrients [17, 18]. Further, spore or pellicle release from biofilms is known to act as a dispersal method in some biofilm forming fungi [19, 20].

In this work, we test the hypothesis that *Z. tritici* spores would be able to survive for extended periods without nutrients, particularly if previously nutrient-replete. We find that, when blastospores are transferred from a rich culture medium to water, a sub-population of remains culturable and virulent for at least 49 days, and we investigate nutrient use and gene expression during this prolonged starvation period.

## Results

### Survival of *Z. tritici* blastospores in water

**Fig. 1:**
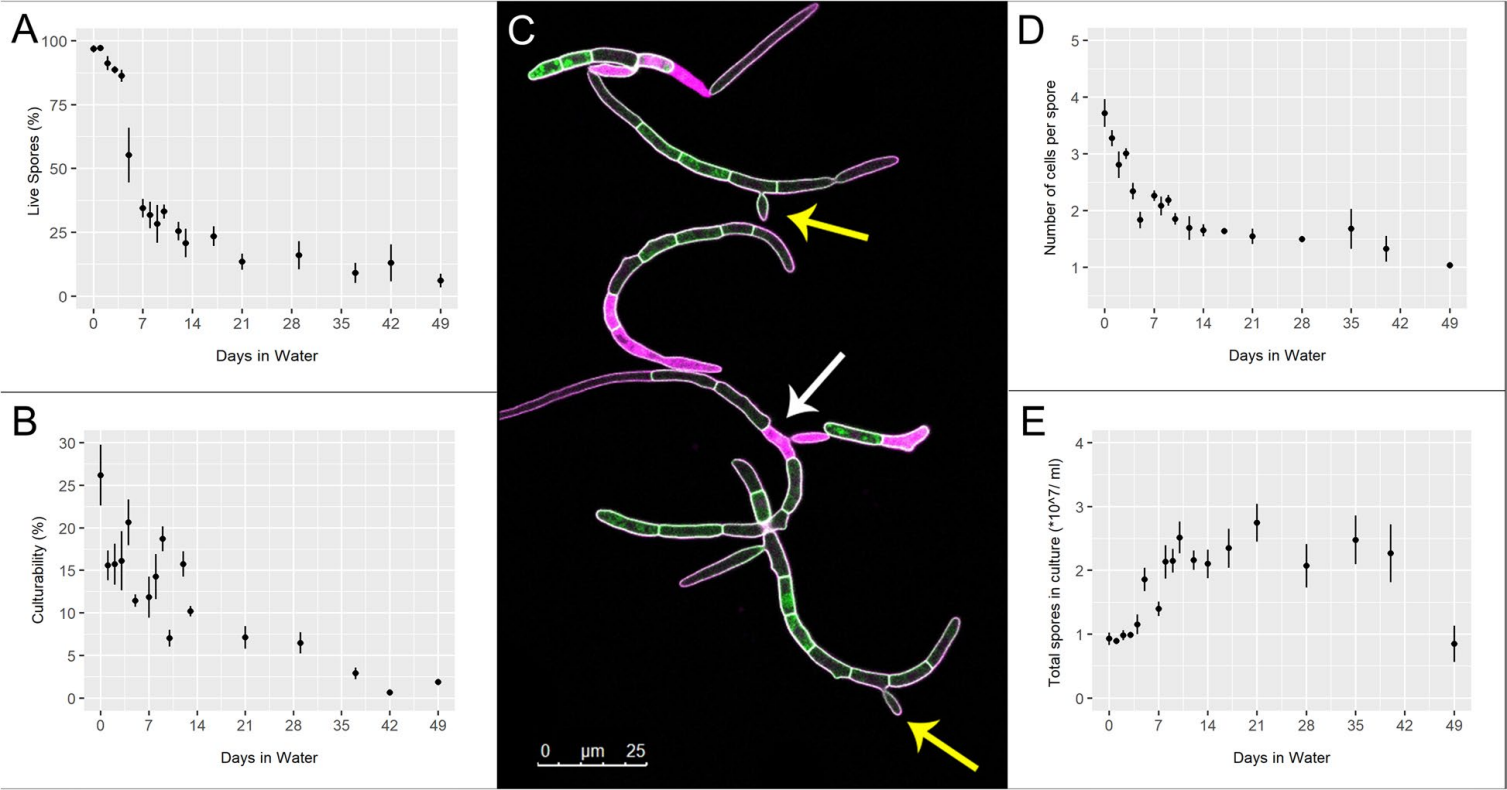
Assessment of *Z. tritici* blastospore populations over 49 days suspended in water. **A** Percentage viable blastospores (spores with at least one live cell; assessed by live/dead staining with 0.05% (w/v) propidium iodide). **B** Percentage culturability—number of colonies from plating 100 blastospores as quantified by haemocytometer. **C** Blastospores expressing cytoplasmic ZtGFP (green) were stained with propidium iodide (pink) after 5 days in water. White arrow highlights an example dead cell; yellow arrows highlight instances of budding growth. **D** Mean number of cells per blastospore. **E** Total blastospore count of populations over time using a haemocytometer. In all experiments, data at each time point are means of counts from 4 independent experiments, each containing at least 4 confocal images totalling at least 100 blastospores (**A**, **D**); or 3 spread plates (**B**) or 3 haemocytometer counts (**E**). Error bars show SE

Following rain-splash dispersal, asexual spores of *Z. tritici* are most likely to land on host or non-host plant surfaces, or soil. They will be suspended in rainwater which may, or may not, include some dissolved nutrients from the cirrus [21–24]. This is likely to represent a low-nutrient environment. To determine how well *Z. tritici* spores can survive in such environments, we investigated their survival in autoclaved MilliQ water, representing the most extreme version of these conditions, starvation (Fig. 1). Spores were assessed in multiple ways over a 49-day period. Live/dead staining with propidium iodide revealed that the proportion of live spores (defined as a fungal structure having at least one live constituent cell; Fig. 1A, C; Additional file 1: Fig. S1) fell slowly for the first 5 days and then declined very rapidly between days 5 and 7. However, from day 7 onwards, the rate of decline slowed again, with the proportion of live spores remaining steady after around 20 days at approximately 10%. Visual inspection shows that there is an increased number of dead cells per spore and more visible lysed cells at the end of the experimental time course (Additional file 1: Fig. S1). Percentage culturability (defined as the ability of a spore to form a colony on YPD agar; Fig. 1B) was lower than the % live cells at all time points, but followed a similar pattern, with over 2% of spores still culturable after 49 days in water. Spore size, measured by the mean number of cells in each spore, followed a hollow curve with rapid initial decline slowing over time and reaching the minimum of one cell per spore by day 49 (Fig. 1D). Strikingly, the total number of spores per ml of suspension increased over the first ~ 7 days, reaching a plateau of over twice the starting number, which was maintained until around day 40 (Fig. 1E). This increase suggests either budding is occurring or that larger spores can split. Instances of budding were seen (Fig. 1C; yellow arrows), as were instances of death in non-end-cells (Fig. 1C; white arrows), which might represent points at which spores may later split into two or more. Exemplar images taken after 5 and 42 days in water are given in Additional file 2: Fig. S2. Collectively, these results show that ~ 10% of blastospores remained viable and culturable after extended submergence water.

### Nutrient stores utilised by *Z. tritici* blastospores in water

**Fig. 2:**
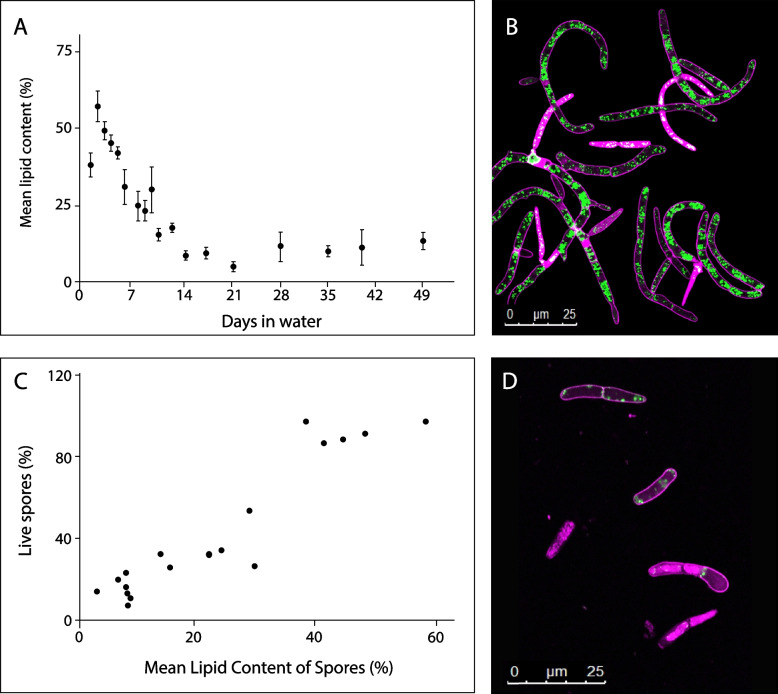
Blastospores show depletion of lipids over time when suspended in water. **A** Average percentage area of spores stained by BODIPY® 493/503. Lipid content of fungal blastospore population is calculated as the percentage of image filled with green fluorescence (lipid granules), divided by area of image representing fungal tissue within the bounds of plasma membranes stained by propidium iodide (PI). Data are means of assessments from 4 independent experiments, each containing at least 4 confocal images of spore populations for each time point. **B** Example image of PI (pink) and BODIPY®-stained cells (green) after 0 days in suspended in water. BODIPY® stain appears white in dead cells where the pink and green signals are overlaid. **C** Correlation between lipid content and spore viability. Experimental data for spore viability taken from a 49-day time spore-viability course shown in Fig. 1A. Data show positive correlation of 0.94 (Pearson’s product-moment correlation, *t* = 5.23, df = 16, *P* < 0.00005). **D** Example image of PI and BODIPY®-stained cells after 49 days in suspended in water. The higher proportion of dead cells, flooded with PI, can be seen in this image, as well as the large reduction in BODIPY®-stained lipid granules

**Fig. 3:**
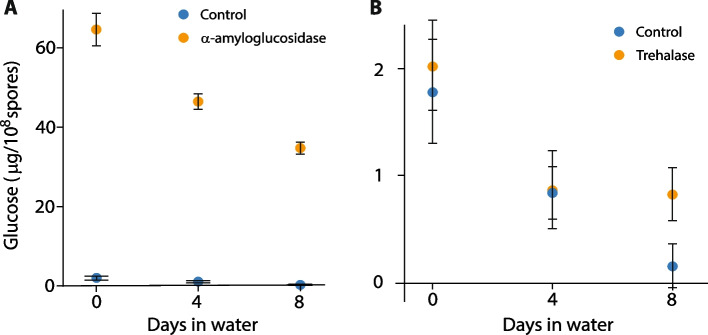
Assessment of glycogen (**A**) and trehalose (**B**) concentrations over time in *Z. tritici* cells suspended in water. Blastospores were treated with either *Aspergillus niger* α﻿-amyloglucosidase (**A**) or porcine trehalase (**B**). Glucose liberated from each reaction was assayed using a Glucose (GO) Assay Kit. Controls were not treated with either enzyme and so reflect the native free glucose content of *Z. tritici* cells. Sample optical density was measured at 540 nm using a spectrophotometer and compared against prepared glucose standards. Data are means of two experiments, each containing three replicate samples (2 samples on day 8 only). Circles represent overall means; small crosses show means from each experiment

To gain insight into the possible energy sources used by *Z. tritici* to survive this extended period of starvation, we investigated the use of three common fungal storage compounds, lipids [25], glycogen [26] and trehalose [26, 27]. Lipid droplets were visualised using the fluorescent lipid stain BODIPY®493/503 and the percentage of spores occupied calculated (Fig. 2). Mean lipid content decreased rapidly for the first ~ 14 days, and then remained at a low plateau of ~ 10% for the rest of the time course (Fig. 2A). There is a clear visual difference in the amount of BODIPY® staining in cells at the beginning and end of the time course (Fig. 2B, D) and a strong positive correlation (Pearson’s product-moment correlation = 0.94) between the percentage of live spores and the mean spore lipid content (Fig. 2C). In addition, we measured the glycogen and trehalose content of spores at days 0, 4 and 8 after suspension in MilliQ. These measurements were carried out by measuring glucose release following enzymatic breakdown of the two compounds; control samples without enzyme treatment therefore measured native free glucose in *Z. tritici* cells. Native free glucose was low (< 2 μg/ml per 10^8^ spores) prior to starvation and fell to < 0.2 μg/ml per 10^8^ spores after 8 days of starvation (Fig. 3), although this decrease is non-significant (ANOVA, *P* = 0.52). α﻿-amyloglucosidase treatment yielded increases in measured glucose up to 64.4 μg/ml per 10^8^ spores on day 0. The concentration of glucose liberated by α-amyloglucosidase appeared to fall over the time course of the experiment (this reduction was non-significant; ANOVA, *P* = 0.11) but always remained significantly greater than the glucose concentration of controls (*t*-tests, *P* = 0.0014, 0.005 and 0.002, respectively, for days 0, 4 and 8), indicating the presence of glycogen in *Z. tritici* cells grown on YPD agar (Fig. 3A). The glucose concentration in samples treated with trehalase showed no significant difference from the controls on any day (*t*-tests, *P* = 0.45, 0.49 and 0.21, respectively, for days 0, 4 and 8), indicating that *Z. tritici* does not produce trehalose as a storage compound during growth on YPD agar (Fig. 3B). Taken together, these results indicate that lipids are likely the main energy source for *Z. tritici* spores during starvation.

### Longevity is maintained with reduced nutrient utilisation during starvation under dry conditions

**Fig. 4:**
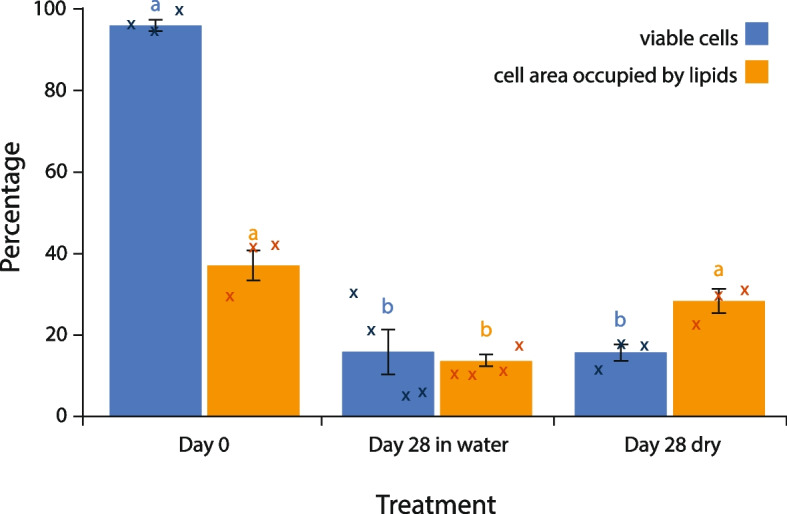
Survival of *Z. tritici* spores suspended in water and allowed to dry out is comparable to spore suspensions maintained in water, but lipid depletion is reduced. Blastospores were suspended in sterile MilliQ water as before. Spore suspensions were either maintained in water or spread onto sterile Petri dishes and allowed to dry under sterile air before the dishes were sealed. Percentage viable spores (PI staining) and lipid content (BODIPY® staining) are shown for cell suspensions at day 0 and after 28 days after suspension in water with or without subsequent drying. Values are means of 3 independent experiments, each comprising a minimum of four technical replicates, and error bars show SE. Significant differences in ANOVAs with Tukey’s simultaneous comparisons are indicated by different letters above bars. Letters apply only to the data whose colour they match. Small crosses indicate means from each experiment

After *Z. tritici* spores are dispersed from pycnidia by rain-splash, a potential source of abiotic stress is drying. To determine whether drying would reduce the longevity or alter the rate at which lipids are depleted by *Z. tritici* spores during starvation, cells were suspended in sterile MilliQ water as previously but then the suspension was allowed to dry on a sterile plastic surface. At 7-day intervals, dried cells were resuspended in 2 ml sterile distilled water and 100 μl aliquots plated onto YPD agar. Resuspended cells were found to be culturable for at least 56 days (Additional file 3: Fig. S3). Live/dead staining and lipid content measurements were undertaken for cells resuspended after 28 days, using the same methods as for cells suspended in water (Fig. 4). While viability declines over this time period (ANOVA, *P* < 0.0001), no difference in viability is seen between wet and dry cells at day 28 (Tukey’s simultaneous comparisons, *P* = 0.49). Significant differences in lipid content were found between samples (ANOVA, *P* = 0.001), reflecting the same decline in lipids in spores in aqueous suspension as reported above, but there was no significant decline in lipid content for cells in dried suspensions (Tukey’s simultaneous comparisons, *P* = 0.0006 and *P* = 0.07, respectively). This suggests that *Z. tritici* spores do not metabolise lipids—or do so at a much reduced rate—when exposed to both starvation and drying, compared to starvation alone.

### Surviving spores retain the ability to cause disease on wheat following starvation

**Fig. 5:**
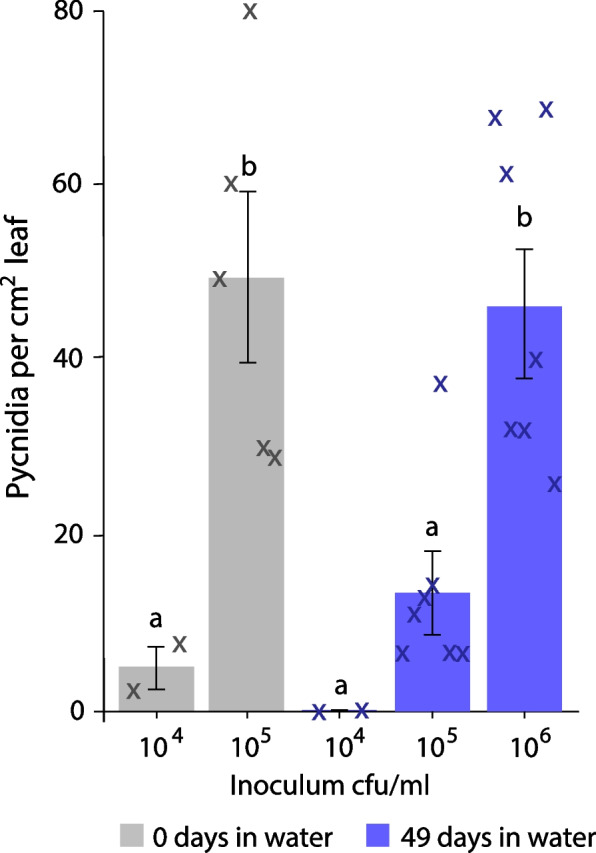
Virulence of *Z. tritici* spores after 49 days in water is comparable to that of fresh spore suspensions. Blastospores were suspended in sterile MilliQ water and maintained for 49 days as before, or suspended from YPD plates immediately prior to use. Spore suspensions were adjusted to 10^4^, 10^5^ or 10^6^ spores per ml and inoculated onto wheat leaves. Pycnidia were enumerated after 28 days. Values are means of two (for 10^4^) or seven (for 10^5^ and 10^6^) independent experiments, in which at least 15 leaves were analysed, and error bars show SE. Different letters above bars indicate significant differences in ANOVA (*P* < 0.0001) with Tukey’s simultaneous comparisons. Small crosses show data points from individual experiments

Following the discovery that *Z. tritici* spores can survive at least 49 days of starvation, with or without water, the virulence of these surviving starved spores was assessed. Wheat leaves were inoculated with spores either taken directly from YPD plates or maintained for 49 days in MilliQ water, as before. A range of inoculum densities were used in order to compare virulence more accurately [28] and to account for the 90% drop in spore viability seen after 49 days in water. Inoculation with starved spores led to the production of pycnidia on the wheat leaves, indicating that virulence can be maintained. The number of pycnidia produced by starved inoculum was equivalent to that produced by fresh spore suspensions at 10 × lower spores/ml (Fig. 5). When the drop to 10% viability in the starved spore population is taken into account, this equates to an equivalent rate of pycnidium formation per viable spore in the inoculum.

#### Survival and virulence of blastospores in soil

**Fig. 6:**
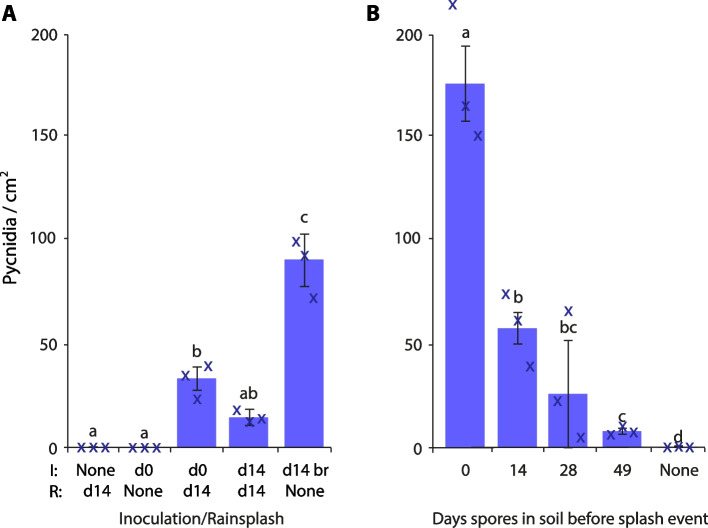
*Z. tritici* spores remain virulent after 49 days in soil and can infect plants during rain-splash events. **A** Wheat was grown in autoclaved soil. Either when the first shoots emerged (d0) or when the plants were 14 days old (d14), the soil was inoculated with *Z. tritici* blastospores (5 ml of 10^6^ spores/ml per cell of a 24-cell tray). Rainfall was simulated on d14 by watering from a height of 2 m at a rate of 4 l of sterile distilled water per 24-cell tray from a rose head watering can. Negative controls without *Z. tritici* or without rainfall were included, and brush inoculation (br) on d14 was used as a positive disease control. **B** Wheat was subjected to simulated rainfall at the indicated intervals after the soil had been inoculated with *Z. tritici* blastospores (5 ml of 10^7^ spores/ml per cell of a 24-cell tray). Uninoculated soil (‘None’) was used as a negative control. In both **A** and **B**, pycnidia per cm^2^ of leaf was calculated for the cotyledon, first and second leaf 28 days after the rain-splash or brush inoculation. Values are means of three independent experiments and error bars show SE. Different letters above bars indicate significant differences in ANOVA with Tukey’s simultaneous comparisons. Small crosses indicate the individual experiment means

To increase the field-relevance of these findings, the survival and subsequent virulence of *Z. tritici* blastospores in soil was assessed. Spreading samples of autoclaved, inoculated soil onto YPD plates yielded *Z. tritici* colonies for at least 49 days. To test the virulence of spores ‘stored’ in soil, two experiments were conducted. Firstly, wheat seeds were planted in autoclaved soil which was inoculated with *Z. tritici* blastospores (5 ml of 10^6^ spores/ml per pot) and subsequently subjected to simulated rainfall. Neither simulated rain-splash from uninoculated soil nor the growing of plants in inoculated soil without rain-splash yielded pycnidia, as expected. However, rain-splash from soil inoculated either on the day of the simulated rainfall or 14 days prior led to pycnidiation (Fig. 6A), albeit at a significantly lower rate per cm^2^ than seen with the positive control of brush inoculation with a 10^6^ spores/ml blastospore suspension (Fig. 6A; ANOVA with Tukey’s simultaneous comparisons; *P* < 0.0001). There was no significant difference in the amount of pycnidia produced by spores that had been in soil for 14 days vs 0 days (Fig. 6A; ANOVA with Tukey’s simultaneous comparisons; *P* = 0.08).

Secondly, soil was inoculated with *Z. tritici* blastospores (5 ml of 10^7^ spores/ml per pot) and seeds were sown periodically such that plants were 14 days old at various times after the inoculation of the soil. Rainfall was simulated at these time points and plants assessed for the development of pycnidia (Fig. 6B). In line with the decline of spore viability seen in water, these rain-splash events yielded progressively fewer pycnidia as the spores aged (ANOVA: *P* < 0.0001; Fig. 6B), but, again in line with previous results, pycnidia were formed even when the simulated rain occurred 49 days after the soil was inoculated. Thus, as in water, spores survive and remain virulent in soil for at least 49 days.

### Changes in gene expression during suspension in water

We hypothesised that the ability of *Z. tritici* spores to survive extended periods of starvation while suspended in water must be underpinned by significant changes in gene expression. To test this and to gain insight into the nature of these changes, we carried out RNAseq to compare gene expression in *Z. tritici* blastospores on YPD to blastospores grown on YPD and then suspended in MilliQ water. Given that the virulence of *Z. tritici* blastospores has been shown to decline rapidly in water [14], we further hypothesised that changes in gene expression would be rapid. We therefore included samples of cells harvested at 1, 4 and 24 h post-suspension in water in this RNAseq experiment. To reveal gene expression associated with longer term starvation, we also included samples of cells harvested after 7 days in water. It proved challenging to extract RNA from later time points due to a large drop in RNA content of cell suspensions, suggesting that global gene expression might be much lower in starved cells, as well as reflecting the high proportion of cells already shown to be dead by this time (only ~ 35% of spores contained a live cell and only ~ 12% were culturable after 7 days in water—see Fig. 1A and B).

**Fig. 7:**
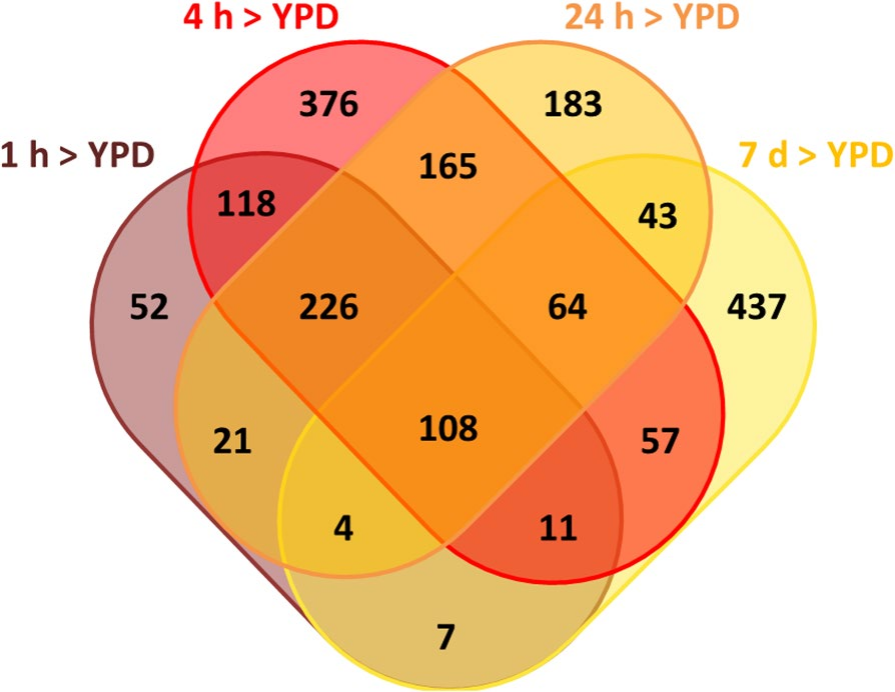
Venn diagram showing numbers of genes upregulated uniquely or in common with specific other time points. Venn diagram produced using http://bioinformatics.psb.ugent.be/webtools/Venn/

**Fig. 8:**
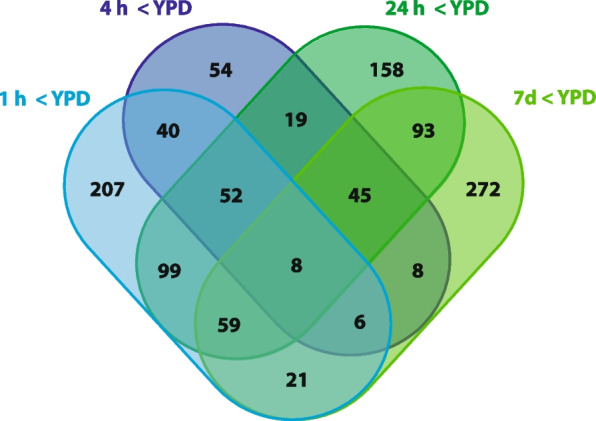
Venn diagram showing numbers of genes downregulated uniquely or in common with specific other time points. Venn diagram produced using http://bioinformatics.psb.ugent.be/webtools/Venn/

**Fig. 9:**
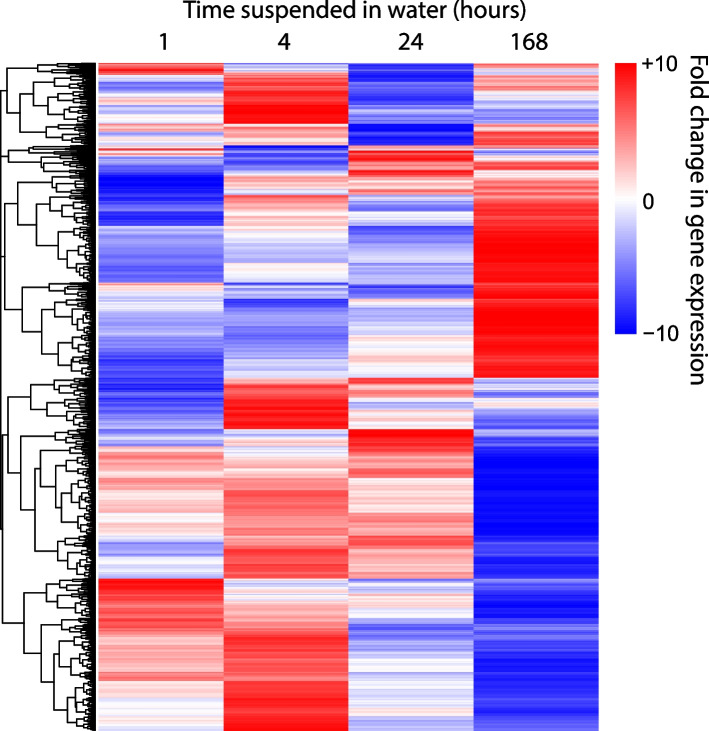
Heatmap of differentially expressed genes at 1, 4, 24 h or 7 days in water, compared to expression on YPD agar. Produced using SR plot [29]

We compared the expression of genes at each of these starvation time points to their expression under nutrient-replete conditions (YPD agar). There are 108 genes upregulated and 8 downregulated in common at all time points; conversely, there are genes uniquely upregulated or downregulated at each time point (Figs. 7 and 8). The largest number of uniquely upregulated genes (437) is seen after 7 days in water, and the least (52) at the end of the first hour. Similarly, the largest number of downregulated genes (272) is seen after 7 days in water. The lowest number of uniquely downregulated genes is seen following 4 h of starvation in water. A heatmap of differentially expressed genes shows that the changes in gene expression after 7 days are distinct from those at the earlier time (Fig. 9).

### Genes upregulated uniquely at 1 h

Interestingly, genes uniquely upregulated at this time are found only on core chromosomes. The most upregulated genes (Table S1; Additional file 4: Supplementary Tables) include a β-glucanase, membrane proteins and a cell wall galactomannoprotein; enriched GO terms (Table 1) include those for cell wall localisation (GO:0005618; *P* = 0.016) and cell wall organisation (GO:0071555; *P* = 0.0096 and GO:0071554; *P* = 0.0128). In addition, metabolic pathways relating to peptidoglycan synthesis and cross-linkage, as well as epoxysqualene and lineolate biosynthesis, are significantly over-represented among the upregulated genes (Table S2; Additional file 4: Supplementary Tables).

**Table 1:** GO term enrichment among genes uniquely upregulated after 1 h in water. GO enrichment carried out using tools in FungiDB [30]. Threshold for inclusion in table *P* < 0.02

GO ID	GO Term	Genes in background with this term	Genes uniquely up-regulated at 1 h	Fold enrichment	Odds ratio	*P*-value	Gene IDs
GO:0030312	external encapsulating structure	5	1	62.24	82.33	0.0160	ZTRI_1.1034
GO:0005618	cell wall	5	1	62.24	82.33	0.0160	ZTRI_1.1034
GO:0004455	ketol-acid reductoisomerase activity	1	1	311.18	Infinity	0.0032	ZTRI_1.395
GO:0004506	squalene monooxygenase activity	1	1	311.18	Infinity	0.0032	ZTRI_3.484
GO:0003913	DNA photolyase activity	1	1	311.18	Infinity	0.0032	ZTRI_4.76
GO:0007623	circadian rhythm	1	1	311.18	Infinity	0.0032	ZTRI_6.443
GO:0048511	rhythmic process	1	1	311.18	Infinity	0.0032	ZTRI_6.443
GO:0045229	external encapsulating structure organization	3	1	103.73	164.72	0.0096	ZTRI_1.1034
GO:0071555	cell wall organization	3	1	103.73	164.72	0.0096	ZTRI_1.1034
GO:0071554	cell wall organization or biogenesis	4	1	77.79	109.79	0.0128	ZTRI_1.1034

### All genes upregulated at 1 h

There are 547 genes upregulated after 1 h in water. The most highly upregulated genes (Table S3; Additional file 4: Supplementary Tables) relate mainly to stress responses; they include a HSP-like protein (ZTRI_6.402) with homology to *Aspergillus* Hsp30/Hsp42, which is implicated in the response to a number of stresses including heat, antifungals and oxidative stress [31–33], two genes encoding major facilitator superfamily transporters (ZTRI_8.748, ZTRI_11.277). ZTRI_8.748 has homology to *Candida albicans* gene C6_02480W_A, known to be part of the core stress response in that organism [34] and to tetracycline efflux transporter genes in other fungi, while ZTRI_11.277’s homologue in *C. albicans*, C1_11480W_A, is a stress-induced phosphate transporter. Enriched GO terms in this gene set include unfolded protein binding and protein folding (GO:0051082, GO:0006457; *P* = 0.0017, *P* < 0.0001), heat shock protein binding and chaperone binding (GO:0031072, 0051087; *P* = 0.012, 0.019) (Table 2.).

**Table 2:** GO term enrichment among all genes upregulated after 1 h in water. GO enrichment carried out using tools in FungiDB [30]. Threshold for inclusion in table *P* < 0.02

GO ID	GO Term	Genes in background with this term	Genes up-regulated at 1 h	Fold enrichment	Odds ratio	*P*-value	Gene IDs
GO:0016491	oxidoreductase activity	666	47	1.54	1.72	0.0013	ZTRI_1.1103, 1.153, 1.1625, 1.1653, 1.197, 1.2111, 1.218, 1.388, 1.391, 1.395, 1.494, 1.498, 1.542, 11.103, 12.262, 13.91, 2.1028, 2.632, 3.484, 3.534, 3.917, 4.167, 4.168, 4.169, 4.436, 4.462, 4.643, 4.646, 4.769, 5.122, 5.295, 5.347, 5.473, 5.646, 5.763, 6.437, 6.50, 6.645, 7.211, 7.373, 7.432, 7.458, 7.494, 8.259, 8.269, 8.435, 9.626
GO:0055114	oxidation-reduction process	600	42	7	1.53	0.0028	ZTRI_1.1103, 1.153, 1.1653, 1.197, 1.218, 1.391, 1.395, 1.494, 1.498, 1.542, 10.487, 11.103, 12.262, 13.91, 2.1028, 2.632, 3.484, 3.534, 3.917, 4.167, 4.168, 4.169, 4.436, 4.462, 4.643, 4.646, 5.295, 5.347, 5.473, 5.646, 5.763, 6.437, 6.50, 6.645, 7.373, 7.432, 7.458, 7.494, 8.259, 8.269, 8.435, 9.626
GO:0051082	unfolded protein binding	20	5	5.46	7.08	0.0017	ZTRI_4.535, 5.501, 5.654, 5.783, 8.716
GO:0031072	heat shock protein binding	4	2	10.93	21.02	0.0118	ZTRI_ 5.501, 5.783
GO:0046906	tetrapyrrole binding	102	10	2.14	2.32	0.0176	ZTRI_1.153, 1.864, 4.167, 5.122, 6.437, 7.432, 7.458, 8.259, 8.269, 9.626
GO:0020037	heme binding	102	10	2.14	2.32	0.0176	ZTRI_1.153, 1.864, 4.167, 5.122, 6.437, 7.432, 7.458, 8.259, 8.269, 9.626
GO:0004806	triglyceride lipase activity	5	2	8.74	14.01	0.0190	ZTRI_1.253, 1.6239
GO:0004857	enzyme inhibitor activity	5	2	8.74	14.01	0.0190	ZTRI_12.381, 6.275
GO:0051087	chaperone binding	5	2	8.74	14.01	0.0190	ZTRI_1.1194, 3.651
GO:0060590	ATPase regulator activity	5	2	8.74	14.01	0.0190	ZTRI_1.1194, 3.651
GO:0006457	protein folding	26	8	30.8	6.73	0.0000	ZTRI_1.1194, 4.535, 5.501, 5.654, 5.783, 8.689, 8.716, 9.277
GO:0046351	disaccharide biosynthetic process	3	2	66.7	14.57	0.0061	ZTRI_6.64, 7.164
GO:0005992	trehalose biosynthetic process	3	2	66.7	14.57	0.0061	ZTRI_6.64, 7.165
GO:0009312	oligosaccharide biosynthetic process	3	2	66.7	14.57	0.0061	ZTRI_6.64, 7.166
GO:0005984	disaccharide metabolic process	5	2	40	8.74	0.0190	ZTRI_6.64, 7.167
GO:0009311	oligosaccharide metabolic process	5	2	40	8.74	0.0190	ZTRI_6.64, 7.168
GO:0005991	trehalose metabolic process	5	2	40	8.74	0.0190	ZTRI_6.64, 7.169
GO:0006631	y acid metabolic process	13	3	23.1	5.04	0.0192	ZTRI_12.262, 4.169, 4.769

There were also enriched functions relating to metabolism, including lipase activity (GO:0004806, *P* = 0.0190), fatty acid metabolic process (GO:0006631; *P* = 0.0019) and several closely related GO terms attached to two genes, ZTRI_6.64 and ZTRI_7.164—trehalose phosphatase and trehalose phosphatase synthase. In addition, metabolic pathways relating to fatty acid β-oxidation, trehalose biosynthesis and fatty acid salvage are significantly over-represented among the upregulated genes (Table S4; Additional file 4: Supplementary Tables).

### Genes upregulated uniquely at 7 days

There are 732 genes upregulated after 7 days in water compared to YPD. Of these, 437 are not upregulated at any other time point. Highly upregulated (Table S5; Additional file 4: Supplementary Tables) and enriched GO terms (Table 3.) include those with functions relating to oxidation/reduction (GO: 0016705, 0016491, 0055114; *P* < 0.0005), iron ion binding and haem binding (GO: 0005506, 0020037, 0046914; *P* < 0.0005, < 0.0005, 0.001). All of the genes associated with enriched GO terms involved in haem binding or tetrapyrrole binding are CYPs (cytochrome P450 oxidases). These and other over-represented GO terms were associated with a range of genes with annotations that only arose once in the results. To better understand any commonalities in function between these genes, their PFAM domains were investigated and the number of times each domain occurs in gene products associated with significantly enriched GO terms (Bonferroni corrected *P* < 0.05) is shown, along with PFAM domain descriptions, in Table S6 (Additional file 4: Supplementary Tables). The most common domain is PF0067 (cytochrome p450; 15 gene products), followed by PF00264 (tyrosinase; 4 gene products). Also among this gene set were 11 fungal specific zinc cluster proteins, fungal-specific regulators of metabolism and stress response (Table S7; Additional file 4: Supplementary Tables).

**Table 3:** GO terms significantly enriched in genes upregulated uniquely after 7 days in water. Enrichment analysis carried out using tools in FungiDB [30]. *P* value threshold for inclusion is 0.02

GO ID	GO Term	Genes in background with this term	Genes uniquely up-regulated at 7d	Fold enrichment	Odds ratio	*P*-value	Gene IDs
GO:0016705	oxidoreductase activity, acting on paired donors, with incorporation or reduction of molecular oxygen	126	20	4.31	5.3800	0.0000	ZTRI_1.1603, 1.1735, 1.1783, 1.1784, 1.187, 1.1950, 1.834, 10.145, 10.489, 11.126, 12.61, 2.10, 2.1087, 3.103, 4.395, 6.233, 6.595, 7.340, 7.9, 8.549
GO:0005506	iron ion binding	113	18	4.32	5.3500	0.0000	ZTRI_1.1603, 1.1735, 1.1783, 1.1784, 1.187, 1.834, 10.145, 10.489, 11.126, 12.61, 2.1138, 2.570, 3.103, 4.395, 6.595, 7.340, 7.9, 8.549
GO:0046906	tetrapyrrole binding	102	16	4.26	5.2100	0.0000	ZTRI_1.1603, 1.1735, 1.1783, 1.1784, 1.187, 1.834, 10.145, 10.489, 11.126, 12.61, 3.103, 4.395, 6.595, 7.340, 7.9, 8.549
GO:0020037	heme binding	102	16	4.26	5.2100	0.0000	ZTRI_1.1603, 1.1735, 1.1783, 1.1784, 1.187, 1.834, 10.145, 10.489, 11.126, 12.61, 3.103, 4.395, 6.595, 7.340, 7.9, 8.549
GO:0016491	oxidoreductase activity	666	48	1.96	2.3700	0.0000	ZTRI_1.1215, 1.1603, 1.1735, 1.1783, 1.1784, 1.187, 1.1950, 1.724, 1.834, 10.145, 10.35, 10.489, 10.546, 11.126, 12.28, 12.61, 13.227, 1.253, 2.10, 2.1015, 2.1087,2.1138, 2.57, 2.570, 2.646, 2.751, 3.103, 3.1037, 3.875, 4.120, 4.395, 4.484, 4.613, 4.675, 4.724, 5.424, 6.2333, 6.247, 6.595, 7.139, 7.340, 7.431, 7.9, 8.140, 8.548, 8.549, 9.334, 9.542
GO:0048037	cofactor binding	428	33	2.09	2.4200	0.0000	ZTRI_1.1215, 1.1603, 1.1735, 1.1783, 1.1784, 1.187, 1.1950, 1.724, 1.834, 10.145, 10.35, 10.489, 10.546, 11.126, 12.28, 12.61, 2.10, 2.1015, 2.1087, 3.103, 4.395, 4.484, 4.613, 6.247, 6.595, 7.340, 7.9, 8.4458, 8.549, 9.334, 9.542, 9.475, 8.749, 11.273, 11.184
GO:0046914	transition metal ion binding	420	30	1.94	2.1900	0.0003	ZTRI_1.1162, 1.1603, 1.1735, 1.1783, 1.1784, 1.187, 1.834, 1.2033, 2,1138, 2.282, 2.370, 2.563, 2.570, 2.851, 3.103, 3.875, 4.395, 5.64, 5.757, 6.595, 7.340, 7.421, 7.578, 7.9, 8.549, 10.145, 10.489, 11.126, 11.185, 12.61
GO:0046872	metal ion binding	562	31	1.5	1.6200	0.0134	ZTRI_1.1162, 1.1603, 1.1735, 1.1783, 1.1784, 1.187, 1.2033, 1.834 2,1138, 2.282, 2.370, 2.563, 2.570, 2.851, 3.103, 3.875, 4.395, 5.64, 5.757, 6.595, 7.340, 7.421, 7.578, 7.9, 8.549, 10.145, 10.489, 11.126, 11.185, 12.61, 13.308
GO:0043169	cation binding	565	31	1.49	1.6100	0.0144	ZTRI_1.1162, 1.1603, 1.1735, 1.1783, 1.1784, 1.187, 1.2033, 1.834 2,1138, 2.282, 2.370, 2.563, 2.570, 2.851, 3.103, 3.875, 4.395, 5.64, 5.757, 6.595, 7.340, 7.421, 7.578, 7.9, 8.549, 10.145, 10.489, 11.126, 11.185, 12.61, 13.308
GO:0050660	flavin adenine dinucleotide binding	125	10	2.17	2.3400	0.0164	ZTRI_1.1950, 1.724, 10.35, 2.10, 2.1015, 2.1087, 4.484, 4.613, 9.475, 9.542
GO:0055085	transmembrane transport	506	41	2.2	2.6500	0.0000	ZTRI_1.1859, 1.1864, 1.726, 10.85, 2.1016, 2.1018, 2.1168, 2.328, 3.207, 3.224, 3.642, 3.672, 3.731, 3.82, 4.179, 4.204, 4.255, 4.459, 4.621, 4.645, 4.723, 4.879, 5.184, 5.511, 5.516, 5.61, 5.637, 6.6 6.83, 7.15, 7.154, 7.843, 8.278, 8.286, 8.537, 11.146, 11.264, 12.259, 13.195, 13.32, 13.39
GO:0055114	oxidation-reduction process	600	40	1.81	2.0900	0.0001	ZTRI_1.1215, 1.1603, 1.1735, 1.1783, 1.1784, 1.187, 1.1950, 1.724, 1.834, 10.145, 10.35, 10.489, 10.546, 11.126, 12.61, 13.227, 2.10, 2.1015, 2.1087, 2.1138, 2.57, 2.570, 3.103, 3.1037, 3.875, 4.120, 4.395, 4.484, 4.613, 4.724, 5.424, 6.233, 6.247, 6.595, 7.340, 7.9, 8.548, 8.549, 9.334, 9.542
GO:0006810	transport	677	42	1.68	1.9300	0.0004	ZTRI_1.1193, 1.1859, 1.1864, 1.726, 10.85, 2.1016, 2.1018, 2.1168, 2.328, 3.207, 3.224, 3.642, 3.672, 3.731, 3.82, 4.179, 4.204, 4.255, 4.459, 4.621, 4.645, 4.723, 4.879, 5.184, 5.511, 5.516, 5.61, 5.637, 6.6 6.83, 7.15, 7.154, 7.843, 8.278, 8.286, 8.537, 11.146, 11.264, 12.259, 13.195, 13.32, 13.39
GO:0051234	establishment of localization	679	42	1.68	1.9200	0.0004	ZTRI_1.1193, 1.1859, 1.1864, 1.726, 10.85, 2.1016, 2.1018, 2.1168, 2.328, 3.207, 3.224, 3.642, 3.672, 3.731, 3.82, 4.179, 4.204, 4.255, 4.459, 4.621, 4.645, 4.723, 4.879, 5.184, 5.511, 5.516, 5.61, 5.637, 6.6 6.83, 7.15, 7.154, 7.843, 8.278, 8.286, 8.537, 11.146, 11.264, 12.259, 13.195, 13.32, 13.39
GO:0051179	localization	690	42	1.65	1.8800	0.0005	ZTRI_1.1193, 1.1859, 1.1864, 1.726, 10.85, 2.1016, 2.1018, 2.1168, 2.328, 3.207, 3.224, 3.642, 3.672, 3.731, 3.82, 4.179, 4.204, 4.255, 4.459, 4.621, 4.645, 4.723, 4.879, 5.184, 5.511, 5.516, 5.61, 5.637, 6.6 6.83, 7.15, 7.154, 7.843, 8.278, 8.286, 8.537, 11.146, 11.264, 12.259, 13.195, 13.32, 13.39
GO:0051704	multi-organism process	2	2	27.13	Infinity	0.0014	ZTRI_1.1979, 11.307
GO:0016021	integral component of membrane	523	36	1.87	2.1400	0.0001	ZTRI_1.1864, 1.726, 10.85, 2.1016, 2.1018, 2.1138, 2.1168, 2.328, 3.224, 3.41, 3.642, 3.672, 3.731, 4.179, 4.204, 4.255, 4.270, 4.621, 4.645, 4.723, 5.184, 5.511, 5.594, 5.61, 5.637, 5.720, 6.6 6.83, 7.15, 7.154, 8.278, 8.286, 8.537, 11.264, 13.195, 13.32, 13.39
GO:0031224	intrinsic component of membrane	523	36	1.87	2.1400	0.0001	ZTRI_1.1864, 1.726, 10.85, 2.1016, 2.1018, 2.1138, 2.1168, 2.328, 3.224, 3.41, 3.642, 3.672, 3.731, 4.179, 4.204, 4.255, 4.270, 4.621, 4.645, 4.723, 5.184, 5.511, 5.594, 5.61, 5.637, 5.720, 6.6 6.83, 7.15, 7.154, 8.278, 8.286, 8.537, 11.264, 13.195, 13.32, 13.39
GO:0016020	membrane	819	47	1.56	1.7800	0.0009	ZTRI_1.1859, 1.1864, 1.726, 10.85, 2.1016, 2.1018, 2.1138, 2.1168, 2.328, 3.207, 3.224, 3.41, 3.642, 3.672, 3.731, 4.179, 4.204, 4.255, 4.270, 4.459, 4.621, 4.645, 4.723, 4.879, 5.149, 5.184, 5.511, 5.516, 5.594, 5.61, 5.637, 5.720, 6.6 6.83, 7.15, 7.154, 7.843, 8.278, 8.286, 8.537, 11.146, 11.264, 11.307, 12.259, 13.195, 13.32, 13.39
GO:0044425	membrane part	603	37	1.66	1.8700	0.0011	ZTRI_1.1864, 1.726, 10.85, 2.1016, 2.1018, 2.1138, 2.1168, 2.328, 3.207, 3.224, 3.41, 3.642, 3.672, 3.731, 4.179, 4.204, 4.255, 4.270, 4.621, 4.645, 4.723, 5.184, 5.511, 5.594, 5.61, 5.637, 5.720, 6.6 6.83, 7.15, 7.154, 8.278, 8.286, 8.537, 11.264, 13.195, 13.32,

### Genes upregulated at all time points

There are 108 genes that are upregulated at every time point. As for the 7-day time point, GO terms enriched (Table 4.) include tetrapyrrole/haem/iron ion binding (GO:0046906, GO:0020037, GO:0005506), and these, once again, are associated with genes annotated as CYP-450 s. Flavin oxygenases also appear (GO:0016712), along with more general oxidation–reduction GO terms (GO:0016899, GO:0016491, GO:0016705, GO:0055114) as does transmembrane transport (GO:0055085). A number of enriched GO terms also relate to lipid metabolism: fatty acid metabolic process (GO:0006631; *P* = 0.0043), lipid catabolic process (GO:0016042; *P* = 0.0058), fatty acid beta-oxidation (GO:0006635, *P* = 0.015), lipid oxidation (GO:0034440; *P* = 0.015) and fatty acid oxidation (GO:0019395; *P* = 0.015).

**Table 4:** GO terms significantly enriched in genes upregulated at all time points investigated. Enrichment analysis carried out using tools in FungiDB [30]. *P* value threshold for inclusion is 0.02

GO ID	GO Term	Genes in background with this term	Genes up-regulated at all time points	Fold enrichment	Odds ratio	*P*-value	Gene IDs
GO:0046906	tetrapyrrole binding	102	6	7.59	9.2	0.0001	ZTRI_1.153, 4.167, 5.122, 7.432, 7.458, 9.626
GO:0020037	heme binding	102	6	7.59	9.2	0.0001	ZTRI_1.153, 4.167, 5.122, 7.432, 7.458, 9.626
GO:0016491	oxidoreductase activity	666	14	2.71	3.66	0.0003	ZTRI_1.153, 1.194, 1.498, 1.542, 12.262, 2.1028, 4.167, 4.168, 4.169, 4.646, 5.122, 7.432, 7.458, 9.626
GO:0055114	oxidation-reduction process	600	13	2.8	3.69	0.0004	ZTRI_1.153, 1.494, 1.498, 1.542, 12.262, 2.028, 4.167, 4.168, 4.169, 4.646, 7.432, 7.458, 9.626
GO:0055085	transmembrane transport	506	10	2.55	3.09	0.0043	ZTRI_1.1497, 10.200, 11.214, 11.277, 2.813, 3.156, 4.5, 5.179, 8.407, 8.748
GO:0006631	fatty acid metabolic process	13	2	19.85	24.42	0.0043	ZTRI_12.262, 4.169
GO:0048037	cofactor binding	428	9	2.71	3.24	0.0046	ZTRI_1.153, 12.262, 2.1028, 3.830, 4.167, 5.122, 7.432, 7.458, 9.626
GO:0016042	lipid catabolic process	15	2	17.2	20.65	0.0058	ZTRI_12.262, 6.239
GO:0046577	long-chain-alcohol oxidase activity	1	1	129.02	Infinity	0.0078	ZTRI_2.1028
GO:0004584	dolichyl-phosphate-mannose- glycolipid alpha- mannosyltransferase activity	1	1	129.02	Infinity	0.0078	ZTRI_3.314
GO:0005506	iron ion binding	113	4	4.57	5.1	0.0109	ZTRI_1.153, 4.167, 7.432, 7.458, 9.626
GO:0016899	oxidoreductase activity, acting on the CH-OH group of donors	2	1	64.51	131.2	0.0154	ZTRI_2.1028
GO:0070330	aromatase activity	2	1	64.51	131.2	0.0154	ZTRI_7.458
GO:0003997	acyl-CoA oxidase activity	2	1	64.51	131.2	0.0154	ZTRI_12.262
GO:0016712	oxidoreductase activity, acting on paired donors	2	1	64.51	131.2	0.0154	ZTRI_7.458
GO:0006635	fatty acid beta-oxidation	2	1	64.51	131.2	0.0154	ZTRI_12.262
GO:0006777	Mo-molybdopterin cofactor biosynthetic process	2	1	64.51	131.2	0.0154	ZTRI_13.333
GO:0034440	lipid oxidation	2	1	64.51	131.2	0.0154	ZTRI_12.262
GO:0019395	fatty acid oxidation	2	1	64.51	131.2	0.0154	ZTRI_12.262
GO:0019720	Mo-molybdopterin cofactor metabolic process	2	1	64.51	131.2	0.0154	ZTRI_13.333
GO:0016705	oxidoreductase activity, acting on paired donors, with incorporation or reduction of molecular oxygen	126	4	4.1	4.54	0.0157	ZTRI_1.153, 4.167, 7.432, 7.458

### Genes downregulated uniquely at 1 h

The GO terms most enriched among genes downregulated only after 1 h in water included many with roles in primary metabolism—including peptidase activity, mannosidase activity and carbohydrate binding (Table 5.), but the most strongly downregulated genes are all annotated only as encoding predicted or hypothetical proteins (Table S8; Additional file 4: Supplementary Tables). Possible transcription factors in this gene set (Table S9; Additional file 4: Supplementary Tables) include ZTRI_2.860, which has homology to the *amdx* gene in *Aspergillus flavus* and *Magnaporthe oryzae*. *AmdX* regulates the expression of *amdS*, an acetamidase in *Aspergillus nidulans* that allows acetamide to be used as a source of either nitrogen or carbon [35, 36]. The metabolic pathways significantly enriched among these genes are shown in Table S10 (Additional file 4: Supplementary Tables).

**Table 5:** GO terms significantly enriched in genes downregulated uniquely after 1 h in water. Enrichment analysis carried out using tools in FungiDB [30]. *P* value threshold for inclusion is 0.02

GO ID	GO Term	Genes in background with this term	Genes uniquely down-regulated at 1 h	Fold enrichment	Odds ratio	P-value	Gene IDs
GO:0016705	oxidoreductase activity, acting on paired donors, with incorporation or reduction of molecular oxygen	129	9	5.42	6.35	0.0000	ZTRI_1.1068, 1.1950, 1.549, 1.828, 3.31, 5.698, 7.219, 9.359, 10.506
GO:0003824	catalytic activity	2985	55	1.43	2.37	0.0001	ZTRI_1.1068, 1.1244, 1.1310, 1.1376, 1.1387, 1.1605, 1.1759, 1.1822, 1.1950, 1.549, 1.764, 1.828, 2.1193, 2.323, 2.525, 2.63, 2.96, 3.1013, 3.31, 3.368, 4.700, 4.732, 4.95, 5.341, 5.575, 5.698, 6.150, 7.219, 7.234, 7.431, 7.439, 8.140, 8.254, 10.193, 10.212, 10.262, 10.273, 10.375, 10.400, 10.429, 10.494, 10.506, 10.65, 11.122, 12.330, 12.438, 13.174, 13.198, 14.84
GO:0004497	monooxygenase activity	91	7	5.98	6.9	0.0002	ZTRI_1.1068, 1.1950, 1.828, 5.698, 7.219, 9.359, 12.330
GO:0016709	oxidoreductase activity, acting on paired donors, with incorporation or reduction of molecular oxygen, NAD(P)H as one donor, and incorporation of one atom of oxygen	25	4	12.43	15.31	0.0003	ZTRI_1.1068, 1.1950, 5.698, 7.219
GO:0050661	NADP binding	39	4	7.97	9.17	0.0015	ZTRI_1.1068, 1.1950, 7.219, 10.193
GO:0016491	oxidoreductase activity	781	20	1.99	2.35	0.0017	ZTRI_1.1068, 1.1950, 1.549, 1.828, 2.63, 3.31, 4.732, 5.698, 6.150, 7.219, 7.234, 7.431, 8.140, 8.548, 9.359, 10.193, 10.400, 10.494, 10.506, 12.330
GO:0004499	N,N-dimethylaniline monooxygenase activity	21	3	11.1	13.24	0.0023	ZTRI_1.1068, 1.1950, 7.219
GO:0004222	metalloendopeptidase activity	32	3	7.28	8.2	0.0078	ZTRI_2.1193, 2.252, 9.112
GO:0030246	carbohydrate binding	34	3	6.86	7.67	0.0092	ZTRI_1.549, 10.273, 10.429
GO:0004517	nitric-oxide synthase activity	1	1	77.69	Infinity	0.0129	ZTRI_5.698
GO:0004567	beta-mannosidase activity	1	1	77.69	Infinity	0.0129	ZTRI_1.1605
GO:0004767	sphingomyelin phosphodiesterase activity	1	1	77.69	Infinity	0.0129	ZTRI_10.375
GO:0004348	glucosylceramidase activity	1	1	77.69	Infinity	0.0129	ZTRI_3.1013
GO:0090729	toxin activity	1	1	77.69	Infinity	0.0129	ZTRI_1.1979
GO:0006684	sphingomyelin metabolic process	1	1	76.74	Infinity	0.0130	ZTRI_10.375
GO:0048288	nuclear membrane fusion involved in karyogamy	1	1	76.74	Infinity	0.0130	ZTRI_4.159
GO:0000741	karyogamy	1	1	76.74	Infinity	0.0130	ZTRI_4.159
GO:1990456	mitochondrion-endoplasmic reticulum membrane tethering	1	1	76.74	Infinity	0.0130	ZTRI_2.459
GO:0000740	nuclear membrane fusion	1	1	76.74	Infinity	0.0130	ZTRI_4.159
GO:0006685	sphingomyelin catabolic process	1	1	76.74	Infinity	0.0130	ZTRI_10.375
GO:0070096	mitochondrial outer membrane translocase complex assembly	1	1	76.74	Infinity	0.0130	ZTRI_2.459
GO:0090173	regulation of synaptonemal complex assembly	1	1	76.74	Infinity	0.0130	ZTRI_1.709
GO:0060629	regulation of homologous chromosome segregation	1	1	76.74	Infinity	0.0130	ZTRI_1.709
GO:0060631	regulation of meiosis I	1	1	76.74	Infinity	0.0130	ZTRI_1.709
GO:0000742	karyogamy involved in conjugation with cellular fusion	1	1	76.74	Infinity	0.0130	ZTRI_4.159
GO:0006665	sphingolipid metabolic process	15	2	10.23	11.92	0.0158	ZTRI_10.375, 3.1013
GO:0051783	regulation of nuclear division	15	2	10.23	11.92	0.0158	ZTRI_1.709, 1.1244
GO:0005506	iron ion binding	115	5	3.38	3.65	0.0159	ZTRI_1.549, 1.828, 3.31, 6.150, 9.359
GO:0055114	oxidation-reduction process	643	15	1.79	1.99	0.0177	ZTRI_1.1950, 1.549, 1.828, 10.193, 10.400, 10.494, 10.506, 3.31, 5.698, 6.150, 7.219, 7.234, 8.548, 9.359

### All genes downregulated at 1 h in water (vs YPD)

There are a total of 492 genes significantly downregulated after 1 h in water; 34 GO terms are significantly enriched among them, as shown in Table 6.. These encompass a diverse range of functions. The top 10 most downregulated genes (Table S11; Additional file 4: Supplementary Tables) also include transporters, as well as a GFY plasma-membrane protein likely to function as an acetate channel [37]. There are 23 genes likely to represent transcription factors (Table S12; Additional file 4: Supplementary Tables) and over 100 metabolic pathways that are over-represented in this gene list (Table S13; Additional file 4: Supplementary Tables).

**Table 6:** GO terms significantly enriched among all genes downregulated after 1 h in water. Enrichment analysis carried out using tools in FungiDB [30]. *P* value threshold for inclusion is 0.02

GO ID	GO Term	Genes in background with this term	Genes down-regulated at 1 h	Fold enrichment	Odds ratio	*P*-value	Gene IDs
GO:0016705	oxidoreductase activity, acting on paired donors, with incorporation or reduction of molecular oxygen	129	19	3.21	3.77	5.52E-06	ZTRI_1.1068, 1.1950, 1.1989, 1.549, 1.828, 2.61, 3.31, 3.530, 3.697, 4.559, 5.698, 6.497, 7.219, 7.9, 9.359, 10.292, 10.506, 12.284, 13.244
GO:1902222	erythrose 4-phosphate/phosphoenolpyruvate family amino acid catabolic process	3	3	21.78	Infinity	9.59E-05	ZTRI_1.1987,1.1988 5.480
GO:0006559	L-phenylalanine catabolic process	3	3	21.78	Infinity	9.59E-05	ZTRI_1.1987,1.1988 5.480
GO:0044282	small molecule catabolic process	56	10	3.89	4.64	1.94E-04	ZTRI_1.1857, 1.1987,1.1988, 1.950, 1.999, 2.1147, 4.229, 5.480, 7.43, 10.347
GO:0004497	monooxygenase activity	91	13	3.11	3.58	2.34E-04	ZTRI_1.1068, 1.1950, 1.1989, 1.828, 3.530, 4.559, 5.698, 7.219, 7.553, 9.359, 10.292, 12.284, 12.330
GO:0033961	cis-stilbene-oxide hydrolase activity	9	4	9.68	16.84	4.57E-04	ZTRI_1.671, 7.632, 9.323, 13.198
GO:0016803	ether hydrolase activity	9	4	9.68	16.84	4.57E-04	ZTRI_1.671, 7.632, 9.323, 13.198
GO:0020037	heme binding	113	14	2.7	3.04	6.12E-04	ZTRI_1.1989, 1.828, 2.61, 3.16, 3.31, 3.697, 6.289, 6.497, 7.43, 7.9, 9.359, 10.236, 12.284, 13.244
GO:0046906	tetrapyrrole binding	114	14	2.67	3.01	6.69E-04	ZTRI_1.1989, 1.828, 2.61, 3.16, 3.31, 3.697, 6.289, 6.497, 7.43, 7.9, 9.359, 10.236, 12.284, 13.244
GO:0016801	hydrolase activity, acting on ether bonds	10	4	8.71	14.03	7.34E-04	ZTRI_1.671, 7.632 ,9.323, 13.198
GO:0016709	oxidoreductase activity, acting on paired donors, with incorporation or reduction of molecular oxygen, NAD(P)H as one donor, and incorporation of one atom of oxygen	25	6	5.23	6.68	7.52E-04	ZTRI_1.1068, 1.1950, 3.530, 4.559, 5.698, 7.219
GO:0050660	flavin adenine dinucleotide binding	129	15	2.53	2.83	7.77E-04	ZTRI_1.1068, 1.1950, 2.226, 3.31, 3.530, 3.547, 4.559, 5.401, 6.113, 7.219, 7.234, 7.248, 9.263, 9.542, 12.168
GO:0016209	antioxidant activity	49	8	3.56	4.14	1.56E-03	ZTRI_1.1068, 3.16, 4.732, 5.527, 6.289, 10.236, 11.121, 12.423
GO:0050661	NADP binding	39	7	3.91	4.63	1.74E-03	ZTRI_1.1068,1.1950, 3.530, 4.599, 7.219, 9.396, 10.193
GO:0003884	D-amino-acid oxidase activity	2	2	21.78	Infinity	2.10E-03	ZTRI_3.547, 9.542
GO:0004499	N,N-dimethylaniline monooxygenase activity	21	5	5.18	6.59	2.19E-03	ZTRI_1.1068, 1.1950, 3.530, 4.559, 7.219
GO:0005506	iron ion binding	115	13	2.46	2.73	2.23E-03	ZTRI_1.1989, 1.549, 1.828, 2.61, 3.31, 3.697, 6.150, 6.497, 7.9, 8.175, 9.359, 12.284, 13.244
GO:0022857	transmembrane transporter activity	520	38	1.59	1.73	2.51E-03	ZTRI_1.1826, 1.1974, 1.1978 1.726, 2.1147, 2.313, 2.517, 2.576, 3.205, 3.747, 4.505, 4.895, 4.9, 5.283, 5.43, 5.671.1, 5.701, 5.793, 6.140, 6.591, 7.365, 7.545, 8.240, 9.17, 9.170, 9.220, 9.245, 9.29, 10.549, 11.141, 11.205, 11.510, 12.285, 12.35, 12.90, 13.151, 13.357, 13.56
GO:0016684	oxidoreductase activity, acting on peroxide as acceptor	42	7	3.63	4.23	2.71E-03	ZTRI_1.1836, 3.16, 4.732, 5.527, 6.289, 10.236, 11.121
GO:0005215	transporter activity	533	38	1.55	1.68	3.86E-03	ZTRI_1.1826, 1.1974, 1.1978 1.726, 2.1147, 2.313, 2.517, 2.576, 3.205, 3.747, 4.505, 4.895, 4.9, 5.283, 5.43, 5.671.1, 5.701, 5.793, 6.140, 6.591, 7.365, 7.545, 8.240, 9.17, 9.170, 9.220, 9.245, 9.29, 10.549, 11.141, 11.205, 11.510, 12.285, 12.35, 12.90, 13.151, 13.357, 13.56
GO:0016831	carboxy-lyase activity	25	5	4.36	5.27	4.91E-03	ZTRI_3.429, 4.55, 4.700, 4.77, 9.34
GO:0016620	oxidoreductase activity, acting on the aldehyde or oxo group of donors, NAD or NADP as acceptor	26	5	4.19	5.02	5.86E-03	ZTRI_1.999, 4.389, 4.780, 6.152, 9.549
GO:0018576	catechol 1,2-dioxygenase activity	3	2	14.52	41.83	6.11E-03	ZTRI_6.150, 8.175
GO:0019114	catechol dioxygenase activity	3	2	14.52	41.83	6.11E-03	ZTRI_6.150, 8.175
GO:0016702	oxidoreductase activity, acting on single donors with incorporation of molecular oxygen, incorporation of two atoms of oxygen	17	4	5.12	6.47	6.45E-03	ZTRI_1.1987, 5.480, 6.150, 8.175
GO:0019842	vitamin binding	88	10	2.47	2.72	6.74E-03	ZTRI_1.549, 3.492, 4.55, 4.77, 5.399,5.438, 6.84, 9.34, 10.494, 13.282
GO:0004601	peroxidase activity	38	6	3.44	3.96	7.10E-03	ZTRI_1.1836, 3.16, 4.732, 6.289, 10.236, 11.121
GO:0016701	oxidoreductase activity, acting on single donors with incorporation of molecular oxygen	28	5	3.89	4.58	8.12E-03	ZTRI_1.1987, 5.480, 6.150, 7.533, 8.175
GO:0016811	hydrolase activity, acting on carbon-nitrogen (but not peptide) bonds, in linear amides	29	5	3.75	4.39	9.45E-03	ZTRI_2.1147, 4.229, 4.431, 6.315, 7.22
GO:0051213	dioxygenase activity	30	5	3.63	4.21	1.09E-02	ZTRI_1.1987, 5.480, 6.150, 7.533, 8.175
GO:0015293	symporter activity	4	2	10.89	20.91	1.19E-02	ZTRI_5.793, 9.220
GO:0008199	ferric iron binding	4	2	10.89	20.91	1.19E-02	ZTRI_6.150, 8.175
GO:0004371	glycerone kinase activity	4	2	10.89	20.91	1.19E-02	ZTRI_10.347, 5.442
GO:0016903	oxidoreductase activity, acting on the aldehyde or oxo group of donors	32	5	3.4	3.9	1.43E-02	ZTRI_1.999, 4.389, 4.780, 6.152, 9.549

### Genes downregulated after 7 days in water (vs YPD)

There are a total of 512 genes significantly downregulated after 7 days in water; 272 are uniquely downregulated at this time point, including 21 probable transcription factors (Table S14; Additional file 4: Supplementary Tables). Twenty-nine GO terms significantly are enriched among them (Table 7.). The metabolic pathways significantly enriched among these genes are mostly related to primary metabolic functions such as fatty acid or amino acid biosynthesis or degradation (Table S15; Additional file 4: Supplementary Tables).

**Table 7:** GO terms significantly enriched among all genes downregulated after 7 days in water. Enrichment analysis carried out using tools in FungiDB [30]. *P* value threshold for inclusion is 0.02

GO ID	GO Term	Genes in the bkgd with this term	Genes in result with this term	Percent of bkgd genes in result	Fold enrichment	Odds ratio	*P*-value
GO:0004601	peroxidase activity	38	8	21.1	11.52	15.32	3.02E-07
GO:0016684	oxidoreductase activity, acting on peroxide as acceptor	42	8	19	10.42	13.51	6.85E-07
GO:0016209	antioxidant activity	49	8	16.3	8.93	11.19	2.35E-06
GO:0004553	hydrolase activity, hydrolyzing O-glycosyl compounds	130	10	7.7	4.21	4.81	1.19E-04
GO:0016798	hydrolase activity, acting on glycosyl bonds	139	10	7.2	3.94	4.47	2.07E-04
GO:0016491	oxidoreductase activity	781	26	3.3	1.82	2.1	1.50E-03
GO:0022857	transmembrane transporter activity	520	19	3.7	2	2.24	2.56E-03
GO:0005215	transporter activity	533	19	3.6	1.95	2.18	3.38E-03
GO:0102250	linear malto-oligosaccharide phosphorylase activity	1	1	100	54.72	Infinity	1.83E-02
GO:0004657	proline dehydrogenase activity	1	1	100	54.72	Infinity	1.83E-02
GO:0004557	alpha-galactosidase activity	1	1	100	54.72	Infinity	1.83E-02
GO:0046558	arabinan endo-1,5-alpha-L-arabinosidase activity	1	1	100	54.72	Infinity	1.83E-02
GO:0102499	SHG alpha-glucan phosphorylase activity	1	1	100	54.72	Infinity	1.83E-02
GO:0000987	cis-regulatory region sequence-specific DNA binding	1	1	100	54.72	Infinity	1.83E-02
GO:0008184	glycogen phosphorylase activity	1	1	100	54.72	Infinity	1.83E-02
GO:0004645	1,4-alpha-oligoglucan phosphorylase activity	1	1	100	54.72	Infinity	1.83E-02
GO:0005975	carbohydrate metabolic process	218	12	5.5	3.01	3.38	5.83E-04
GO:0055085	transmembrane transport	531	20	3.8	2.06	2.33	1.35E-03
GO:0006865	amino acid transport	17	3	17.6	9.66	11.79	3.35E-03
GO:0046942	carboxylic acid transport	29	3	10.3	5.66	6.34	1.54E-02
GO:0015849	organic acid transport	29	3	10.3	5.66	6.34	1.54E-02
GO:0044419	interspecies interaction between organisms	11	2	18.2	9.95	12.13	1.63E-02
GO:0009405	pathogenesis	11	2	18.2	9.95	12.13	1.63E-02
GO:1902274	positive regulation of (R)-carnitine transmembrane transport	1	1	100	54.72	Infinity	1.83E-02
GO:1902269	positive regulation of polyamine transmembrane transport	1	1	100	54.72	Infinity	1.83E-02
GO:1902272	regulation of (R)-carnitine transmembrane transport	1	1	100	54.72	Infinity	1.83E-02
GO:1902267	regulation of polyamine transmembrane transport	1	1	100	54.72	Infinity	1.83E-02
GO:0006820	anion transport	57	4	7	3.84	4.16	1.99E-02

### Comparison of transcriptomic changes in water with those seen in other low-nutrient environments

**Fig. 10:**
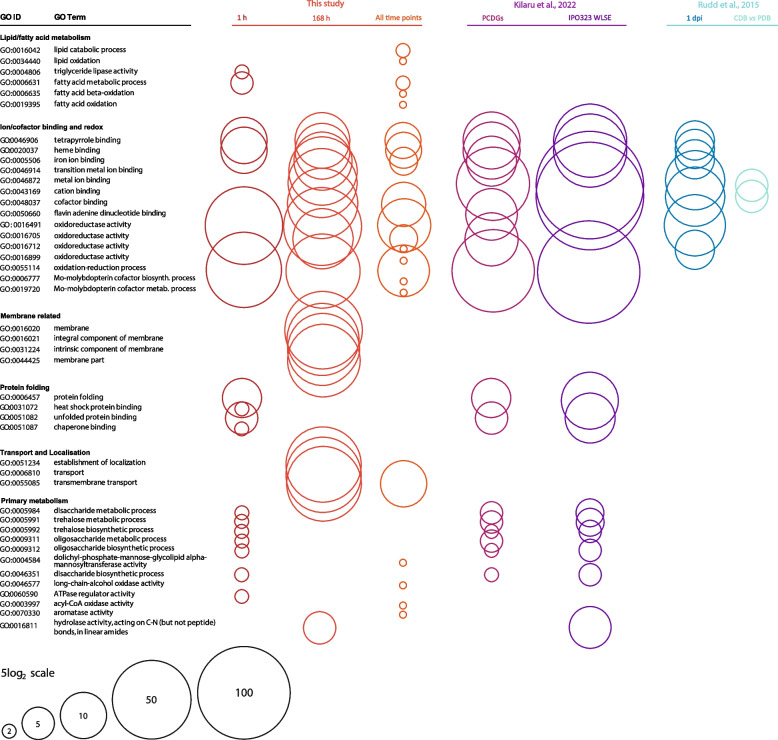
Comparison of numbers of genes related to GO terms enriched in the lists of genes upregulated in this and studies with related, low nutrient conditions. GO terms enriched in the lists of genes upregulated at either 1 h, 7 days or all time points in this study are shown on the left. Circle size represents the number of genes in the gene list associated with each GO term. Also shown are the numbers of genes associated with the same GO terms in other studies, if that GO term is also enriched in the relevant gene list. Studies and conditions included are as follows: Rudd et al. [38]—genes upregulated in cells at 1 day post-inoculation onto wheat leaves vsin vitro growth in CDB, and in cells grown in CDB (low nutrient) for 5 days vs PDB (high nutrient) for 3 days; Kilaru et al. [39]—genes identified as pan-strain core dimorphism genes upregulated in hyphae, and genes upregulated in cells grown for 2–3 days on minimal media + wheat leaf surface extract vs controls without extract

**Fig. 11:**
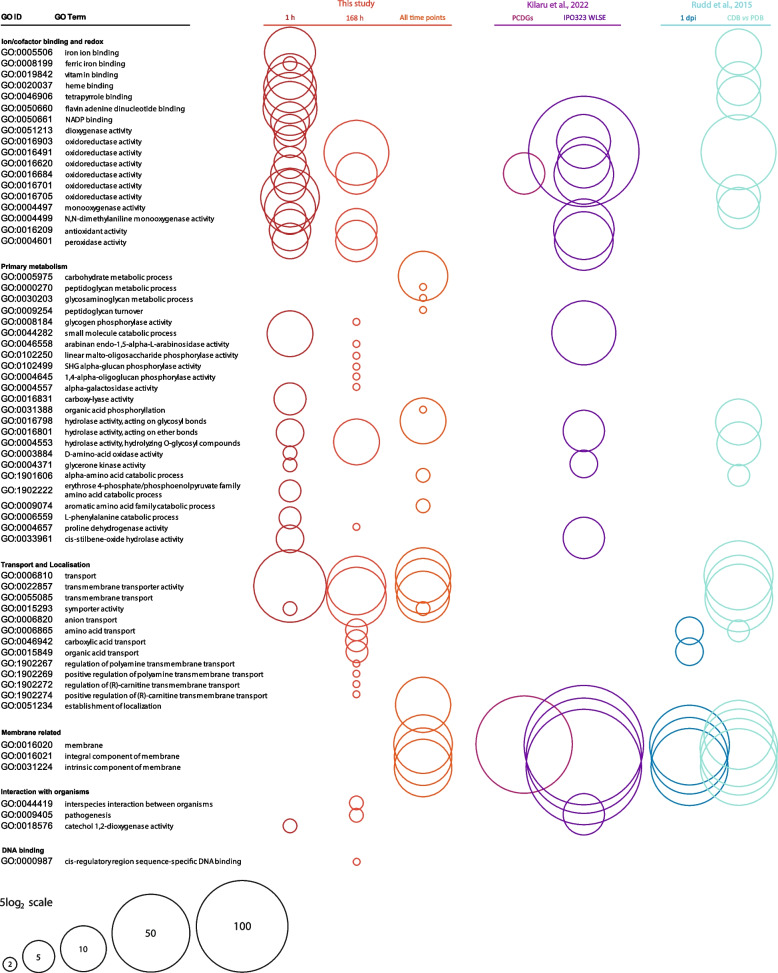
Comparison of numbers of genes related to GO terms enriched in the lists of genes downregulated in this and studies with related, low nutrient conditions. GO terms enriched in the lists of genes downregulated at either 1 h, 7 days or all time points in this study are shown on the left. Circle size represents the number of genes in the gene list associated with each GO term. Also shown are the numbers of genes associated with the same GO terms in other studies, if that GO term is also enriched in the relevant gene list. Studies and conditions included are as follows: Rudd et al. [38]—genes downregulated in cells at 1 day post-inoculation onto wheat leaves vsin vitro growth in CDB, and in cells grown in CDB (low nutrient) for 5 days vs PDB (high nutrient) for 3 days; Kilaru et al. [39]—genes identified as pan-strain core dimorphism genes downregulated in hyphae, and genes downregulated in cells grown for 2–3 days on minimal media + wheat leaf surface extract vs controls without extract

We compared the changes in gene expression that we observed with those seen in related studies. Rudd et al. [38] compared gene expression in cells on either the rich potato dextrose broth (PDB) or the lower nutrient Czapek-Dox broth (CDB), as well as 24 h after inoculation onto a wheat leaf. Kilaru et al. [39] identified genes differentially regulated during the switch to hyphal growth across all isolates of *Z. tritici* investigated, which they termed pan-strain core dimorphism genes (PCDGs). Conditions found to induce hyphal growth and thus alterations in PCDG expression included low nutrient availability (minimal media) and the addition of host cues to the growth medium in the form of wheat leaf surface extracts (WLSEs). Using published lists of differentially expressed genes in those studies [38, 39], we carried out GO term analysis in FungiDB [30]. We then compared the numbers of genes associated with GO terms enriched in these gene lists with those associated with GO terms enriched in our lists of differentially expressed genes at 1 h or 7 days in water, or at all time points in this study. Figure 10 shows the GO terms enriched among upregulated genes at any of the time points in our study, along with the number of genes associated with each term at each time point, in CDB [38], 24 h after inoculation onto the wheat leaf surface [38], in PCDGs [39], or in response to WLSE [39]. Figure 11 shows the same information but for GO terms enriched among downregulated genes in each case. There are clear commonalities between the genes and GO terms that respond to each of the tested conditions, and also differences. Shared GO terms among upregulated genes are largely associated with redox, cofactor binding and primary metabolism, with the greatest similarities appearing between our 1 h time point and the conditions tested by Kilaru et al. [39]. For downregulated genes, the shared GO terms are largely associated with redox, cofactor binding, transport, membranes and primary metabolism, with our 1 h time point most closely resembling WLSE [39] and 7 days having most in common with the CDB condition tested by Rudd et al. [38].

## Discussion

### Long-term survival and maintenance of virulence in the absence of external nutrients has implications for our understanding of the life cycle and population genetics of *Zymoseptoria tritici*

In this work, we have demonstrated that the blastospores of *Z. tritici* are able to survive for long periods without external nutrition. Previously, blastospores grown on YPD have been shown to be similar to pycnidiospores with respect to histology—including indistinguishable cell walls in TEM [5], virulence [40] and response to osmotic shock [14]. Further, pycnidiospores are extruded from pycnidia in a gel-like cirrus. This mucilaginous cirrus is hydrophobic and thought to aid in spore dispersal by rain-splash [13]. In *Parastagonospora nodorum*, another wheat pathogen with rain-splash dispersed conidia, the cirrus contains proteins and sugars, provides protection against desiccation and inhibits germination unless diluted to around 10% of its original concentration [15]. Thus, during the process of rain-splash dispersal of pycnidiospores to a new host leaf, the spores undergo a severe alteration in their environment, from extrusion in a protective and nutrient dense cirrus matrix to very significant dilution into rainwater. This process includes the sudden removal of external nutrients and an osmotic shock. Thus, the transition from YPD to water, investigated here, forms a reasonable proxy for dispersion from the cirrus via rain-splash, although it must be interpreted in the light of current uncertainty around whether pycnidiospores have any specific metabolic programme such as dormancy in place prior to landing on a leaf, and also in light of the fact that pycnidiospore populations are likely to be more uniform than lab-grown blastospores. We also note, however, that blastospores are produced on the leaf surface [5, 6] and that this can happen under biofilm-like conditions involving the production of an extracellular matrix [6, 8] (pre-print) [16], whose composition, like cirrus, is likely to be nutrient rich and protective. This population of spores may therefore also experience sudden removal of external nutrients and osmotic shock during rain-splash dispersal.

Growth and survival might be expected to be very limited after suspension of blastospores in distilled (MilliQ) water, and we have previously seen that culturability and virulence of suspended blastospores begins to fall within minutes [14]. However, we determined that some blastospores within a population remained alive for at least 49 days (7 weeks). Our conditions were very stringent—in the field, spores will have access to at least as many, or more, nutrients as in these experiments. Thus, the ability to survive starvation for at least 49 days is likely to have relevance under field conditions. In the UK, a period of 7 weeks will often be sufficient to span the intercrop period for wheat [41]. Following dispersal, spores are subjected to additional stresses while on the leaf surface, including a risk of desiccation. However, our data indicate that desiccation does not increase spore death rates over a period of 28 days, which reduces the likelihood that such stresses invalidate the long-term spore survival that we propose here. In line with this, Chaloner et al. [42] showed that after a small drop in viability associated with the drying process, proportion of viable spores was not greatly affected by 4 h of dryness. Moreover, we show here that spore survival in soils is comparable to that seen in water. This is important both because wheat plants or stubble will not necessarily be available post-harvest, depending on the management of the field, and because soils retain more moisture and absorb UV light, mitigating the affects of non-starvation stresses. In addition, soil may be carried between fields on farm equipment and may thus be a mechanism for introducing *Z. tritici* spores into fields in which wheat is about to be sown. We also demonstrate that, once adjusted for the drop in viability associated with long-term storage, spores stored in either water or soil for 49 days retain their virulence, and that infection of wheat seedlings could occur as a result of rain-splash dispersal of 49-day-old spores from soil. Taken together, these findings raise the possibility that soil-borne, asexual pycnidiospores, as well as wind-blown, sexual ascospores, can act as primary inoculum for outbreaks of Septoria tritici blotch. This may be relevant to disease management.

Coupled with the production of blastospores in planta by virulent [5] and avirulent [6] *Z. tritici*, the long-term survival of blastospores in soils and low-nutrient environments such as non-host leaves provides a route by which genotypes less able to infect a given crop may remain in the population and potentially contribute genes to later generations via sexual reproduction or vegetative fusion. This has potential impact on the best strategies for managing the emergence of strains virulent on previously resistant wheat cultivars or resistant to fungicides. The ability of *Z. tritici* blastospores to survive desiccation also raises the possibility of aerial dispersal, for which this ability is essential. Like epiphytic bacteria, leaf surface fungi can become airborne [43, 44], providing an alternative route to dispersal. We note that if this were to occur with *Z. tritici*, for example following epiphytic blastosporulation or from leaf surface biofilms [5, 6, 8] (pre-print) our data suggest that desiccated spores would not use their lipid supplies, meaning that if rained out of the atmosphere onto a suitable host, they would still contain enough lipids to support colonisation. This possibility, to our knowledge, has never been explored in *Z. tritici*, whose ascospores are considered responsible for wind-blown dispersal [45, 46].

### Use of lipid stores and changes to primary metabolism likely underpin survival in water

The data presented here indicate that *Z. tritici* blastospores rely on stored lipids for energy during starvation. Lipid content was also significantly positively correlated with spore viability. Surprisingly, trehalose and glycogen were present at low concentration and/or not significantly depleted during starvation, although both are known to be important carbohydrate stores in yeasts such as *Saccharomyces cerevisiae* [26]. In *Aspergillus nidulans*, trehalose is a major storage compound in spores and is rapidly metabolised following germination, with conidial viability in storage rapidly lost in trehalose biosynthesis mutants [27]. However, in many fungi, trehalose is mainly produced under stressful conditions such as starvation [47]; a lack of trehalose in *Z. tritici* blastospores raised under optimal conditions on YPD is consistent with these findings. However, GO terms and metabolic pathways associated with trehalose synthesis were enriched in the set of genes upregulated after an hour in water. Additional functions of trehalose are reported to include temperature, oxidative and osmotic stress tolerance and signalling, as well as the virulence of several phytopathogens [27, 48–51], while trehalose and trehalose-6-phosphate concentrations can be involved in the regulation of glycolysis [26]. Thus, upregulation of trehalose synthesis may be related to survival during starvation in an indirect manner.

Gene expression patterns suggest that the most significant changes undergone by *Z. tritici* cells under starvation are linked to a global downregulation of metabolism. After only 1 h in water, genes involved in primary metabolic functions were already significantly over-represented among downregulated genes. At 1 h, around 10 × more metabolic pathways were significantly over-represented among downregulated genes, compared to upregulated genes and this downregulation of primary metabolism persisted to 7 days. Against this background, lipase activity and pathways relating to fatty acid β-oxidation and salvage are nevertheless upregulated during starvation. This is consistent with the observed depletion of lipid droplets. We hypothesise that the ability of *Z. tritici* to germinate, survive and even proliferate on the low-nutrient leaf surface [4–6, 8] is likely to show strong dependence on prior lipid accumulation in spores.

Dry spores, however, do not use their lipids during starvation, yet retain viability. In *Saccharomyces cerevisiae*, desiccation survival is dependent on lipid breakdown [52]. This suggests that *Z. tritici* may have an as yet un-elucidated mechanism for surviving both starvation and desiccation. Perhaps most likely is a form of metabolic arrest, as seen during the desiccation of micro-colonial fungi on rock surfaces.

### Relationship between cells in an individual during long-term survival

A striking feature of the *Z. tritici* spore population after an extended period of starvation was the observed reduction in average cells per spore, to a minimum of one after 49 days. This appeared to be due in part to budding reproduction, but primarily to the death of cells within the spore, which we propose is likely to lead to spores splitting into smaller units of fewer cells. The increase in total spore counts supports the hypotheses of either budding or breaking, with the decline in this measure from 42 to 49 days indicating that lysis of dead cells is also occurring, and outweighs the rate of budding and splitting once cells no longer have the resources to grow and are already composed of only one cell, which cannot split further. The death of cells within multicellular spores, while the remaining cells remain healthy, raises some important questions about the interactions between the cells of an individual spore. In common with other ascomycete fungi, *Z. tritici* is functionally coenocytic, since the septa which divide the cells are perforated by pores and the cytoplasm is therefore continuous [53, 54]. These pores are kept open in an ATP-dependent manner and are blocked by Woronin bodies to prevent cytoplasmic bleeding if a cell is wounded [55, 56]. This explains how a spore can survive the death of certain cells within it. However, it remains unclear why cell death is triggered in some cells but not others under the same conditions. *Z. tritici* blastospore populations have been shown to behave in a highly asynchronous manner following inoculation onto wheat leaves [4, 9]. The findings here raise the possibility that this asynchronicity also exists at the level of cells within individuals. It is possible that certain cells, due to their status at the moment of suspension in water (for example, their cell cycle stage or nutrient content) are likely to die earlier than others. Cytoplasmic connectivity means that the nutrients within these cells could theoretically be scavenged by their neighbours. In live fungal cells, early endosomes and vacuoles mediate intercellular transport and can pass through septal pores, while transporters can mediate the selective exchange of nutrients between cells, even when septal pores are occluded by Woronin bodies [57–59]. It is thus theoretically possible that nutrients are passed from dying cells into their healthier neighbours. However, against this, it is notable that in the images of cells stained with propidium iodide and BODIPY® in Fig. 2B, lipid bodies are visible in the dead cells, meaning lipid mobilisation and nutrient exchange is not complete prior to cell death. Notably, the mobilisation of lipid stores is thought to be important during the early phases of plant infection in *Z. tritici*, during which nutrient uptake from the host is thought to be extremely limited [1, 38]. There are therefore some parallels between early infection and the starvation conditions imposed here, which are borne out in the observed depletion of stored lipids in this work. However, *Z. tritici* is unusual in that its response to starvation is independent of autophagy [60]. While autophagy is repressed under nutrient-replete conditions in many fungi, including ascomycetes such as *Aspergillus nidulans* [61], this is not the case in *Z. tritici* [60]. Autophagy is often required to recycle nutrients from ageing cells and is often essential for phytopathogen virulence [62, 63], but this is not required for virulence in *Z. tritici* [60]. These findings cast doubt on the idea of nutrient scavenging from dying cells. An alternative hypothesis is that cell lysis, occurring after cell death, liberates cell contents into the growth media and allows their re-uptake by healthy cells, prolonging the life of the population. Such a mechanism would likely be maladaptive in widely dispersed cells, in a population of mixed genotypes or when part of a varied microbiome whose other members could compete for released nutrients. However, in *Z. tritici*, blastosporulation can lead to areas of dense, clonal epiphytic growth occurs on the leaf surface and biofilms containing a mixture of live and dead cells may develop [5, 6, 8]. Thus, there may be field-relevant circumstances in which the re-uptake of lysed cell contents would be almost exclusive to clonal *Z. tritici* cells.

### Other changes in gene expression

The clear differentiation in patterns of gene expression between 7 days in water and the three earlier time points is likely to reflect a the effect of rapid loss of nutrient availability and osmotic shock. In line with this idea, 6 of the 10 GO terms over-represented among genes upregulated uniquely at 1 h are cell wall or membrane related, while GO terms related to sphingolipid metabolism and catabolism are over-represented among downregulated genes. Sphingolipids modulate membrane fluidity, but they have many further roles including in virulence and stress signalling. Their accumulation during starvation, consistent with the downregulation of sphingolipid catabolism seen here, is linked to increased integration of amino acid permeases into the membrane and thus increase amino acid uptake [64, 65], pertinent to starvation. GO terms related to sphingolipid metabolism over-represented among genes downregulated at 1 h also include glucosylceramidase activity. Glucosylceramide is a cell wall component associated with polar growth, hyphal production and germination [66]. The downregulation of glucosylceramidase will reduce the release of ceramides from their glycosylated forms, which may help to reduce ceramide-mediated apoptotic death in response to stress [67]. Other groups of GO terms over-represented among genes downregulated at 1 h are linked to nuclear membrane fusion, karyogamy, and meiosis, suggesting that resource allocation to reproduction is also rapidly reduced on transition to a low-nutrient environment.

At 7 days, by contrast, the majority of over-represented GO terms among upregulated genes are involved in oxidation–reduction processes. PFAM analysis indicated that many of the genes associated with these GO terms were cytochrome P450s (CYPs). CYPs are a superfamily of proteins found all five domains of life and thought to have arisen close to the origin of terrestrial life [68, 69]. They form a multi-component oxygenase system that is involved in a wide range of functions from synthesis of secondary metabolites to detoxification of xenobiotics, adaptation to stress and to new niches [69, 70]. In phytopathogens, they have roles in host-specificity, defence compound detoxification and virulence [69, 71, 72]. In *Saccharomyces cerevisiae* and *Candida glabrata*, CYP functions include fatty acid degradation, as well as synthesis of molecules involved in cell wall and membrane structure [73]. CYPs have previously been shown to be upregulated under starvation stress in fungi and other organisms [74, 75]. Upregulation of CYPs in response to nitrogen limitation has been shown in both ascomycete and basidiomycete fungi [76]. GO terms linked to transport activity and to membrane structure are also over-represented after 7 days, suggesting a possible role for membrane remodelling in starvation tolerance. Over-represented GO terms among downregulated genes were predominantly related to primary metabolism.

### Comparison to related studies

We hypothesised that sudden immersion of blastospores in water following growth on the rich medium, YPD, would provide similar changes to those experienced during the process of a spore being dispersed from cirrus in rain-splash: a large drop in external osmotic potential and nutrient availability. To determine to what extent our results bear this out, we compared changes in gene expression in this study to two other studies: Rudd et al. [38], who compared the transcriptome of *Z. tritici* cells growing on Czapek-Dox broth (CDB), to either potato dextrose broth (PDB) or 1 day after inoculation onto the wheat leaf; and Kilaru et al. [39] who compared the transcriptome of *Z. tritici* grown in minimal medium (MM) with and without the addition of a wheat leaf surface extract (WLSE). These studies thus both provided information about *Z. tritici* gene expression under nutrient limitation, with and without additional host cues (either by inoculation onto a wheat leaf or growth on WLSE). Kilaru et al. also reported genes differentially regulated during hyphal growth across a range of *Z. tritici* isolates (pan-strain core dimorphism genes, PCDGs). Hyphal growth can occur in response to the host but also to temperature stress and nutrient limitation [39]. On comparing GO term enrichment, it is clear that the greatest similarity lies between the 7-day time point in this study and the patterns of gene expression in Kilaru et al.’s PCDGs and WLSE gene lists. Most similarities between GO terms in this study and the WLSE GO terms relate to CYPs. This suggests similar changes in stress response or secondary metabolite synthesis or degradation during growth on WLSE and in starvation. This may indicate that some changes in response to WLSE prepare cells for the starvation that may be endured during prolonged growth on the wheat leaf surface, a possibility which may bear further investigation. The similarity between GO terms enriched among DEGs in the current study and those enriched among Kilaru et al.’s PCDGs suggests that the response to a change in nutrient availability includes functions related to the change to hyphal growth. The most parsimonious explanation here would be that nutrient availability has a greater role in the dimorphism of *Z. tritici* than appreciated. The least similarity overall was between any time point in this study and Rudd et al.’s in planta transcriptome. This suggests that shared function in genes differentially regulated in response to starvation/WLSE/PCDGs is linked to nutrient availability, whereas the response in planta is linked to host defences—in line with previous findings that the plant also responds to the fungus within hours of contact [77, 78].

## Conclusions

In this work, we have demonstrated that *Z. tritici* blastospores can utilise lipid stores in order to survive for long periods, spanning the intercrop period for UK winter wheat, with no external nutrition. This survival occurs both in water and in soil, and although a large proportion of spores do not survive for such an extended period, those that do remain as virulent as spores grown on rich media. Coupled with the epiphytic survival of avirulent isolates [6], this suggests that rain-splash-dispersed inoculum on wheat leaves, volunteer and field margin plants and on soil could survive between crops, regardless of their virulence on the planted wheat cultivar. This implies that early infections of newly sown wheat in September–December [41] could be begun by resident asexual inoculum as well as by wind-blown ascospores. We also showed that *Z. tritici* blastospores tolerate drying out, making it more likely that this long-term survival is feasible under field conditions. Moreover, dried spores did not use their lipid stores, indicating that drying could prolong the life of spores and aid in their distribution. Changes in primary metabolism and transport also underpin survival under starvation, and this may be linked to uptake of nutrients from dead cells, potentially following cell lysis, a mechanism which is likely to be most useful to high densities of cells in biofilms, another survival adaptation. In the very short term, transition from YPD to water also involved changes to cell wall and membrane architecture likely linked to withstanding osmotic stress. Survival of both immersion in water and especially longer-term starvation are linked to the function of cytochrome P450 mono-oxygenases, which are linked to fungal stress tolerance. It is clear from the results presented here that *Z. tritici* blastospores have the posited long-term survival ability, and possible that the changes to primary metabolism, trehalose synthesis and CYP expression represent alterations to cell biochemistry that facilitate this. Taken together, and in combination with both survival on resistant hosts [6] and biofilm formation [8] (pre-print), these findings indicate the *Z. tritici* has a suite of exceptional survival strategies which are likely to be important in understanding its population genetics and the selection pressures on virulence and fungicide resistance traits. In turn, this might support development of novel routes for Septoria leaf blotch control.

## Methods

### Fungal isolates used in this study

All experiments in this study used the commonly studied ‘reference’ isolate, *Z. tritici* IPO323 [2]. IPO323 and IPO323 expressing cytoplasmic ZtGFP [79] were kindly provided by Dr Sreedhar Kilaru and Prof Gero Steinberg.

### Confocal microscopy

Confocal images were obtained using a 63 × oil immersion lens on a Leica SP8 microscope. GFP fluorescence from *Z. tritici* was detected at 510–530 nm using an argon laser with excitation at 500 nm. Images were obtained using LAS-X software and processed as batches using macros written in Adobe Photoshop®.

### Assessment of cells per spore and cell live/dead status using propidium iodide

*Z. tritici* IPO323 was plated from − 80°C glycerol stocks onto YPD agar 7 days before use and maintained at 18 °C before resuspending in sterile MilliQ water at 10^7^ spores/ml. To measure the percentage of spores with at least one live cell, propidium iodide (PI) was added to *Z. tritici* cell suspensions to a final concentration of 0.1% (w/v), and incubated for 30–60 min before aliquots were diluted by 100 × and 10 ml mounted on standard glass microscope slides. Cells were visualised every 24 h using a Leica SP8 confocal microscope, with excitation and detection at wavelengths at 493 and 620–645 nm, respectively. Fields of view were selected at random for imaging. Maximum projections were obtained from image stacks using Leica’s proprietary software. Cells were scored as dead/non-viable if PI was had completely stained the cytoplasm. The total number of cells per blastospore over time was also assessed from these images, as were the total number of spores in suspension.

### Assessing the culturability of water-suspended blastospores

*Z. tritici* IPO323 was plated from − 80 °C glycerol stocks onto YPD agar 7 days before use and maintained at 18°C before resuspending in sterile MilliQ water at 10^7^ spores/ml. Aliquots were then diluted by 10,000 × before spreading 100 μl (~ 100 spores) onto YPD agar. Changes in culturability were assessed by colony counting after 7 days. Culturability was calculated as follows: number of colonies/number of spores plated.

### Measurement of blastospore lipid content

*Z. tritici* IPO323 was plated from − 80°C glycerol stocks onto YPD agar 7 days before use and maintained at 18°C before resuspending in sterile MilliQ water at 10^7^ spores/ml. To measure the percentage spore area occupied by lipids, BODIPY® 493/503 (4,4-difluoro-1,3,5,7,8-pentamethyl-4-bora-3a,4a-diaza-s-indacene, Thermo Fisher, Catalogue number: D3922) was used to stain lipid granules. BODIPY® was stored in DMSO at − 20°C at a concentration of 1 mg/ml and added to *Z. tritici* cell suspensions to a final concentration of 10 mM, along with propidium iodide (PI) counterstain at a final concentration of 0.1% (w/v). After the addition of BODIPY® and PI, blastospore suspensions were visualised by confocal microscopy within 30 min. Images were taken using excitation/emission wavelengths of 493/490–515 nm. Lipid content was assessed as a percentage of spore area by calculating: the total area of the image made up of lipid granules/total area of the images containing fungal tissue × 100. Lipid and spore areas were measured in ImageJ [80] following thresholding for pink (spores) and green (lipid) areas of individual spores by thresholding in HSB colour space and measurement of selected areas (filling selections where necessary for outlined shapes, i.e. live spores).

### Measurement of glycogen and trehalose content

Spore populations were tested over time as per [81]. Briefly, 10^8^ *Z. tritici* IPO323 blastospores were heated at 95°C in 1 M acetic acid and 0.2 M sodium acetate before treatment with either *Aspergillus niger* a-amyloglucosidase (for glycogen), porcine trehalase (for trehalose) or no enzyme (controls). Glucose liberated from each reaction was assayed using a Glucose (GO) Assay Kit (Sigma, GAGO-20). Sample optical density was measured at 540 nm using a spectrophotometer and compared against prepared glucose standards.

### Assessment of blastospore survival in dry conditions

To assess the ability of blastospores to survive periods without water, dry Petri dishes were used. *Z. tritici* IPO323 blastospores were grown on YPD agar for 7 days. Blastospores were then suspended in autoclaved MilliQ water and suspension density estimated using a haemocytometer before plating ~ 1000 spores onto the dry Petri dishes. Dishes were dried for 60 min in a Class II Cabinet before sealing with Parafilm®. Individual plates were re-hydrated every 7 days for a 56-day period by flooding with 2 ml of autoclaved MilliQ water. A sterile spreader was then used to suspend blastospores in solution before a small amount was aliquoted onto YPD and incubated at 18°C under a long-day light cycle. Survival was qualified by assessing plates for *Z. tritici* growth after 7 days. On day 28, aliquots of resuspended blastospores were stained with PI and BODIPY® to assess spore viability and lipid content according to the methods given above.

### Assessment of blastospore survival in soil

For experiments concerning blastospore survival in soil, 25 g of autoclaved John Innes No. 2 soil was added to a Petri dish and flooded with 5 ml of autoclaved MilliQ water. Before assessment, *Z. tritici* blastospores were grown on YPD agar for 7 days. Blastospores were then suspended in autoclaved MilliQ water, estimated using a haemocytometer and pipetted into soil at a rate of 5 ml of 1 × 10^6^ blastospores per ml. Soil plates were sealed with Parafilm® and incubated under standard growth cabinet conditions. Every 7 days, a sterile spreader was placed into the wet soil of an individual plate and spread onto a fresh YPD plate. Plates were incubated at 20°C under a long-day light cycle. Survival was qualified by assessing plates for *Z. tritici* growth after 7 days.

### Wheat cultivation and inoculation

*Triticum aestivum* cv. Consort winter wheat (kindly provided by Nick Palmer of RAGT seeds) was grown on J. Arthur Bower’s John Innes No. 2 Compost. Compost was stored frozen at − 20°C for 3 weeks before use. Two seeds were sown in each cell of a 24-cell modular seed tray containing compost and loosely covered. Trays were then placed into a Whitefurze 38-cm gravel tray and filled with 750 ml distilled water. Plants were placed onto one of the three shelves of a Panasonic MLR-352-PE growth cabinet. A long-day light cycle (16 h of light at 20°C and 8 h of darkness at 15°C) was used with 90% relative humidity, using the maximum light setting (~ 5 × 10 mol m^−2^ s^−1^ at leaf level). Plants were left uncovered for 5 days until growth was visible above the soil and subsequently grown for at least 14, and a maximum of 21 days before use. All plants from which data were combined (i.e. used in repeats of the same experiment) were the same age.

For all foliar applications, blastospores were suspended in autoclaved MilliQ water. Concentrations were estimated by haemocytometer and adjusted to concentrations described in each experiment. Before use, suspensions were supplemented with 0.01% (v/v) Silwet L77. Suspensions were then applied using a paintbrush to the abaxial side of fully expanded leaves, until complete coverage was obtained. Post-inoculation, all wheat plants were stored under standard growth cabinet conditions for 28 days. For the first 5 days, plants were sealed in autoclave bags to maintain maximum humidity.

### Enumeration of pycnidia

Plants were assessed for disease by counting pycnidia per cm^2^ of inoculated leaf tissue. All inoculated leaves (marked at the base at time of inoculation) were assessed for pycnidia, except the cotyledon. Leaves were harvested at 28 dpi, rehydrated in tap water and scanned at high resolution. Scanned images were analysed using colour thresholding in ImageJ [80] to isolate and measure leaf area. Pycnidia were then selected using thresholding for black areas in HSB colour space and further selection of near circular areas in the size range of pycnidia using the ‘analyse particles’ function in ImageJ [80]. ‘Analyse particles’ was used to enumerated selected regions allowing calculation of pycnidia/cm^2^ leaf.

### Soil inoculations and rain-splash plant inoculation experiments

Five millilitres of a 10^6^ or 10^7^ spores/ml blastospore suspension was pipetted into each cell of a 24-cell plant tray, each containing two 14-day-old wheat plants. Blastospores suspensions were allowed to soak into soil for 10 min. To mimic rainfall, trays were watered from a height of 2 m at a rate of 4 l of sterile distilled water per 24-cell tray from a Haws No.14 medium rose head watering can. Disease was assessed as pycnidia per cm^2^ of leaf after 28 days incubation. The cotyledon and first two true leaves were assessed. Three controls were conducted: (i) soil with no blastospores added, (ii) plants grown in blastospore inoculated soil without the rain-splash event and (iii) leaves inoculated by the paint brush method (above).

### RNA extractions

Blastospores were suspended at 1 × 10^7^ spores/μl in 50 ml tubes of MilliQ water. These tubes were shaken vigorously for 10 s every 24 h to maintain oxygenation. At four time points (1 h, 4 h, 24 h and 7 days), four randomly selected 50 ml tubes were centrifuged for 1 min at 2000 × g before freezing pellets in liquid nitrogen. Pellets were subsequently ground to a powder in a pestle and mortar using liquid nitrogen and were not allowed to thaw. Total RNA extractions were carried out using the Qiagen RNeasy Kit (Cat No./ID: 74,903) protocol ‘Purification of Total RNA from Plant Cells and Tissues and Filamentous Fungi’ with an on-column DNase step using Qiagen RNase-Free DNase Set (Cat No./ID: 79254). Samples were finally suspended in 50 µl RNase-free water before storing at − 80°C for analysis.

### RNA sequencing

Samples were prepared using the Illumina TruSeq Stranded mRNA kit and sequenced on a HiSeq 2500 generating between 3.3 and 5.2 million reads per sample. Adapter sequences and low-quality bases (< Q22) were removed using cutadapt [82] version 2.5. Reads before and after trimming were checked for quality using FactQC version 0.10.1 [83], and for common contaminants using FastQ Screen [84]. MultiQC [85] was used to collate and visualise the results. A subset of reads was also checked with BLAST [86] against the NCBI nucleotide database for other contaminants. Results were visualised using Krona [87]. Trimmed reads were aligned using STAR [88] version 2.7.2b to the reference genomes fungiDB-45_ZtriticiIPO323. DESeq2 [89] version 1.24.0 was used to determine differentially expressed genes (DEGs) between time points. DEGs were defined as genes with > log2-fold change in expression and *P* < 0.05.

### Analysis of changes in gene expression

Lists of gene differentially up- and downregulated at each time point were analysed for GO term and metabolic pathways enrichment using the tools available at FungiDB (https://www.fungidb.org). PFAM analysis was carried out by extracting PFAM domains associated with genes of interest manually from FungiDB’s gene pages. A global heatmap of differentially expressed genes was produced using SR plot [29]. Comparison of GO term enrichment between lists of differentially expressed genes produced in this and other studies was carried out by using the GO term enrichment analysis tools at FungiDB with our own gene lists, as before, and with gene lists provided in the data associated with Rudd et al. and Kilaru et al. [38, 39].

## Supplementary Information

Additional file 1: Fig. S1 A As Fig. 1, percentage viable blastospores (spores with at least one live cell; assessed by live/dead staining with propidium iodide (PI)) over time in water; B, C examples of cells sampled and stained with PI after B 5 days in water or C 49 days in water. Yellow arrowheads indicate exemplar dead cells, flooded with PI stain. Green arrowheads indicate exemplar live cells, with staining at plasma membrane only. Red arrowheads indicate matter from dead, lysed cells. Fig. S2 As Fig. 1D, average number of cells per individual blastospore over time in water; B As Fig. 1C, example of cells sampled and stained with PI after 5 days in water; C exemplar blastospores stained with PI after 5 days in water; D exemplar blastospores stained with PI after 42 days in water. Note the reduction in cells per individual blastospore between B/C and D. Arrows in B indicate possible reasons for this change: yellow arrows indicate bud formation, which would initially produce one-cell blastospores (buds); white arrows indicate cells in the middle of a blastospore (i.e. non-terminal cells) which have died, potentially leading to splitting of the blastospore into two smaller individual blastospores. Fig. S3 *Z. tritici* cells survive at least 56 days after aqueous suspensions are allowed to dry. An example plate is shown bearing colonies of *Z. tritici* which arose after cells were suspended in water, and then suspensions allowed to dry completely on a sterile Petri dish surface before sealing the Petri dish for 56 days. Dried cells were then resuspended in water and an aliquot pipetted onto YPD agar and incubated at 18°C for 7 days. Colonies are clearly visible. Raw data underpinning each figure; raw gene expression data.

Additional file 2: Tables S1–S15. Table S1 Top ten genes uniquely upregulated at 1 h; Table S2 Metabolic pathways enriched among genes uniquely upregulated at 1 h; Table S3 Top ten all genes upregulated at 1 h; Table S4 Metabolic pathways enriched among genes upregulated at 1 h; Table S5 Top ten genes uniquely upregulated at 7 days; Table S6 PFAM analysis 7 days; Table S7 Transcription factors upregulated at 7 days; Table S8 Top ten genes uniquely downregulated at 1 h; Table S9 Transcription factors among genes uniquely downregulated at 1 h; Table S10 Metabolic pathways enriched among genes uniquely downregulated at 1 h; Table S11 Top ten all genes downregulated at 1 h; Table S12 Transcription factors among all genes downregulated at 1 h; Table S13 Metabolic pathways enriched among all genes downregulated at 1 h; Table S14 Transcription factors among genes downregulated at 7 days; Table S15 Metabolic pathways enriched among genes downregulated at 7 days.

## Data Availability

Transcriptomes are available in the NCBI SRA: accession numbers SAMN40573238–SAMN405756. All experimental raw data are available as supplementary information with this article (Additional file 5: Raw Data).
